# Vision-Based Collision Warning Systems with Deep Learning: A Systematic Review

**DOI:** 10.3390/jimaging11020064

**Published:** 2025-02-17

**Authors:** Charith Chitraranjan, Vipooshan Vipulananthan, Thuvarakan Sritharan

**Affiliations:** Department of Computer Science and Engineering, University of Moratuwa, Katubedda 10400, Sri Lanka; vipooshan.18@cse.mrt.ac.lk (V.V.); thuvarakan.18@cse.mrt.ac.lk (T.S.)

**Keywords:** collision warning, collision prediction, accident anticipation, accident avoidance, ADAS, vehicle, deep learning, computer vision

## Abstract

Timely prediction of collisions enables advanced driver assistance systems to issue warnings and initiate emergency maneuvers as needed to avoid collisions. With recent developments in computer vision and deep learning, collision warning systems that use vision as the only sensory input have emerged. They are less expensive than those that use multiple sensors, but their effectiveness must be thoroughly assessed. We systematically searched academic literature for studies proposing ego-centric, vision-based collision warning systems that use deep learning techniques. Thirty-one studies among the search results satisfied our inclusion criteria. Risk of bias was assessed with PROBAST. We reviewed the selected studies and answer three primary questions: What are the (1) deep learning techniques used and how are they used? (2) datasets and experiments used to evaluate? (3) results achieved? We identified two main categories of methods: Those that use deep learning models to directly predict the probability of a future collision from input video, and those that use deep learning models at one or more stages of a pipeline to compute a threat metric before predicting collisions. More importantly, we show that the experimental evaluation of most systems is inadequate due to either not performing quantitative experiments or various biases present in the datasets used. Lack of suitable datasets is a major challenge to the evaluation of these systems and we suggest future work to address this issue.

## 1. Introduction

In recent years, collision prediction/warning systems in vehicles have become increasingly important for improving road safety. These systems can predict imminent collisions and alert drivers, giving them time to react and potentially avoid an accident. Therefore, they have become an integral part of advanced driver assistance systems (ADAS) [1]. Depending on the level of autonomy and the situation, prediction of collisions allows the ADAS to take necessary actions such as automatic emergency braking (AEB) and/or steering (AES) if needed. This is crucial in improving the safety of autonomous vehicles as well [2]. Moreover, it is important to maintain vehicle handling stability during emergency maneuvers as they may push the vehicle to its handling limits. Dynamic models and stability control systems have emerged to assist in such situations [3,4].

In recent years, deep learning techniques have improved computer vision remarkably to a level almost reaching human capabilities in visual perception. Therefore, not surprisingly, modern collision warning systems have adopted deep learning-based computer vision techniques. While some collision warning systems rely on a combination of sensors, such as cameras, radar, and lidar [1], others use RGB cameras as the only sensors [2,5]. Even autonomous vehicles are becoming increasingly reliant on deep learning-based computer vision [6,7] with some manufacturers opting to use vision only [8]. Such vision-based systems have the advantage of being less expensive and more compact than systems that use other sensors. This makes vision-based collision warning systems an attractive option for many applications including portable ADAS units that can be fixed on existing vehicles [9]. Their main disadvantage, however, is lower robustness compared to systems that use multiple types of sensors.

Several surveys have reviewed ADAS’s [1,10,11,12]. However, they primarily discuss the various components of ADAS in isolation (e.g., object detection, tracking, road detection, etc.) *not* how these components/algorithms are integrated into a complete collision warning subsystem from receiving sensory input to predicting collisions and issuing warnings. Most of all, they do not review the experimental evaluation of the overall collision warning pipeline. Instead, they focus on the experimental results of each component separately. E.g., the accuracy of object detection, road detection, or traffic sign detection. Although challenging [10], it is crucial to validate how effective these systems are at predicting imminent collisions, issuing timely warnings, and if needed, taking actions to avoid collisions. Therefore, to address this problem, we systematically review how deep learning algorithms have been used in vision-based collision warning systems for vehicles and how their effectiveness has been experimentally evaluated. Following are the research questions we set out to answer through this review.

- What are the deep learning techniques/algorithms used in vision-based collision warning systems and how are they integrated with the various components of a system forming a pipeline from visual perception to the prediction of collisions?
- What are the datasets and experiments used to evaluate such systems with respect to the accuracy and timeliness of the predicted collisions and the inference speed of the system?
- What are the properties of such datasets and experiments, and what are the results achieved?

Recently, Fang et al. [13] have conducted a survey on vision-based traffic accident detection and anticipation. Our work has some overlap with this survey regarding the accident anticipation methods. However, being a systematic review, our work includes many collision warning systems that are not reviewed in [13]. Furthermore, we critically review the experimental evaluation of the included studies, which is an aspect *not* adequately covered in [13] (ref. [13] does provide a review of datasets and evaluation metrics used in traffic accident anticipation but does *not* review the experiments conducted and the results achieved by the reviewed studies).

Note that new car assessment programs (NCAP) such as Euro NCAP [14] and National Highway Traffic Safety Administration (NHTSA) NCAP [15] provide specific protocols for testing crash avoidance features in consumer vehicles, which include forward collision warning (FCW). These tests are used to provide ratings, among other aspects, for the FCW and AEB systems of new vehicles (see [16] for example). Although many modern vehicle models are equipped with FCW and AEB functionality and test ratings are available, the technology and algorithms used in them are usually proprietary and not available in the literature for a review. Furthermore, results of tests conducted in a controlled environment may differ from what can be expected in typical road conditions, especially for vision-based systems for instance, due to occlusions [17] and different weather conditions [15]. On the other hand, false positive alerts that may be triggered in typical traffic conditions can be a nuisance to drivers and, therefore, need to be evaluated [15]. This necessitates further testing.

## 2. Methodology

This section describes the main components of this systematic review. First, the inclusion and exclusion criteria used to select studies for the review are stated. Then, the search and screening strategies are discussed.

### 2.1. Eligibility Criteria

#### 2.1.1. Inclusion Criteria

A study must meet the following criteria in order to be included in this review:

- The method proposed in the study predicts possible collisions between the ego-vehicle and other vehicles, pedestrians, and/or other obstacles.
- It uses monocular or stereo visual input provided by one or more RGB cameras as the sensory input. The camera(s) must be mounted on the ego vehicle.
- It uses deep learning techniques at least for object detection (it may or may not use deep learning in other stages).

#### 2.1.2. Exclusion Criteria

A study would be excluded from the review if any of the following criteria are met.

- The proposed system uses any sensors other than vision. Systems aided by other sensors are beyond the scope of this review.
- The system is primarily designed to predict collision not involving the ego vehicle. I.e., collisions between other vehicles. E.g., systems predicting collision from CCTV, traffic surveillance or drone cameras.
- The proposed system is designed to detect collisions that have already occurred (collision detection systems), not predict/forecast future collisions.

### 2.2. Search Strategy

We searched the following databases to retrieve studies published up to 31 December 2023.

- Scopus
- IEEE Explore Digital Library
- ACM Digital Library
- Google Scholar

The conjunction of the keyword statements listed below was used as the query to search each database:

- Collision keywords: [(collision AND mitigation) OR (time to collision) OR (collision management) OR (collision AND warning) OR (ADAS) OR (Advanced Driver Assistance System) OR (collision avoidance) OR (accident anticipation)]
- Vehicle keywords: [vehicle OR bicycle OR driver]
- Sensor keywords: [vision OR camera OR image OR video]
- Machine learning keywords: [(Deep learning) OR (CNN) OR (convolutional AND neural AND network) OR (LSTM) OR (Long short-term memory) OR (transformer AND neural AND network) OR (attention AND neural AND network) OR (machine learning)]

### 2.3. Screening Strategy

The screening was done in two phases with each unique record independently reviewed by two authors. In the first phase, the titles and abstracts returned as search results were screened for potential relevance according to the inclusion criteria.

**Figure 1:**
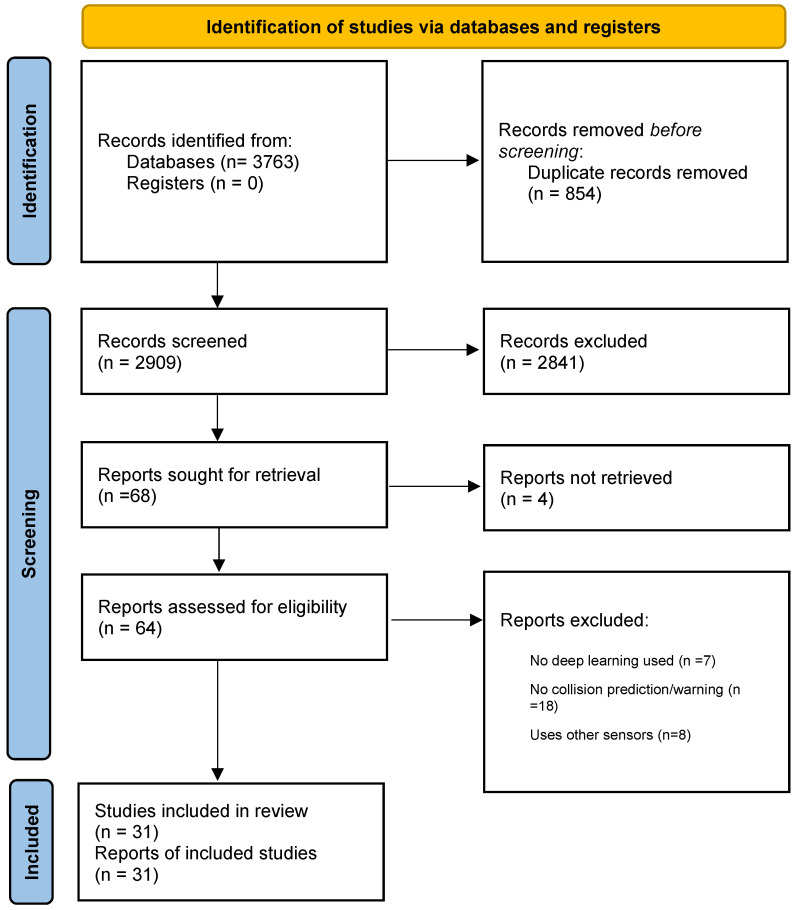
PRISMA [18] flow-diagram for the screening process.

The full text of the publications that passed the first screening phase was sought for retrieval in the second phase. The full text of each retrieved paper was then evaluated by the authors against the inclusion/exclusion criteria, and studies that satisfy all the inclusion criteria and none of the exclusion criteria were included in the review. Thirty-one studies were selected to be reviewed and Figure 1 shows the PRISMA flow-diagram of the screening process. The PRISMA statement is available as Supplementary Material.

The following fields of information, which correspond to the method proposed and its experimental evaluation, were extracted (whenever relevant) from each included study.

- Components in the pipeline (as applicable to a specific study)
  - Object detection method
  - Object tracking method
  - Distance/depth estimation method
  - Trajectory prediction method
  - Threat metric computation or Collision prediction model
  - Hardware platform
- Experimental evaluation
  - Dataset(s) used
  - Experiments conducted
  - Evaluation metrics used and the results achieved

We conducted a risk of bias analysis (ROB) for each included study using the PROBAST tool [19], which we adapted to the type of studies reviewed here (since the input to the studies reviewed here are image sequences without any predefined predictors, questions about predictor variables were ignored). The results of the ROB analysis are available in Supplementary Material. Note that since this is a systematic review, we included all studies that satisfied the inclusion criteria but not the exclusion criteria, regardless of their quality. However, we critically review the experimental evaluation of the studies as a main component of this work, which directly relates to the quality of those studies.

## 3. Results

All the selected papers were read and summarized. The methods proposed in those papers can be mainly categorized into two:

- Methods that compute a threat-metric and then predict collisions based on it (threat metric-based methods).
- Methods that use deep learning to directly predict the probability of a future collision from the input video (collision probability-based methods).

A key distinction between the two types of methods is that collision probability-based methods need a sufficiently large dataset containing videos with accidents and those with normal driving scenes in order to train the deep learning models, whereas the threat metric-based methods do not need to be trained with such accident videos (unsupervised). However, they too need some accident scenarios to test their effectiveness.

**Figure 2:**
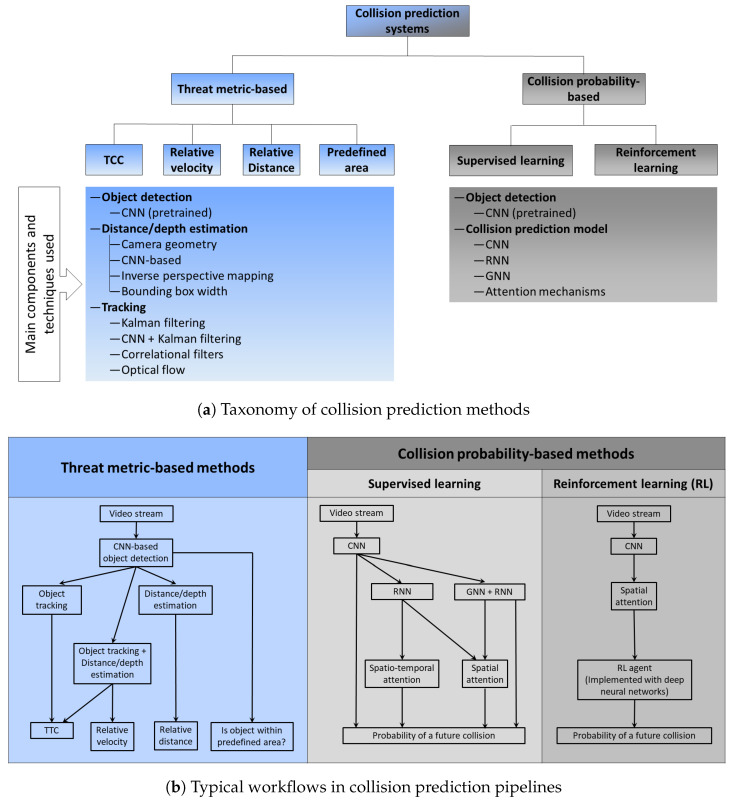
Overview of collision prediction methods: (**a**) Taxonomy and (**b**) workflows. In (**b**), any path of a graph from the root (video stream) to each leaf represents a particular sub-category of methods and the nodes on such a path show the algorithms/techniques/architectures used in that sub-category. Leaf nodes of a graph show the metrics computed by different types of methods. A threshold is applied to the calculated metric to classify whether a collision is likely to occur or not. Abbreviations: CNN, GNN, RNN—convolutional, graph, and recurrent neural network, respectively; TTC—time to collision. Note: Although one-directional arrows are shown from RNN to attention blocks in the graph for supervised learning methods for simplicity, typically there is bi-directional information transfer between them as shown later.

In the rest of this section, we first provide an overview of the collision warning systems proposed in the selected studies, grouped according to the aforementioned two types and further categorized as needed. The main symbols and abbreviations used in this paper are listed in Table 1, while other symbols have been defined in the text as necessary. Figure 2 provides a graphical representation of this categorization, while Table 2 and Table 3 provide a summary of the components used in different systems. The overview covers the main components from data input to the prediction of a possible collision. We then review the datasets and the experiments used to evaluate these systems.

### 3.1. Threat Metric-Based Collision Warning Pipelines

**Figure 3:**
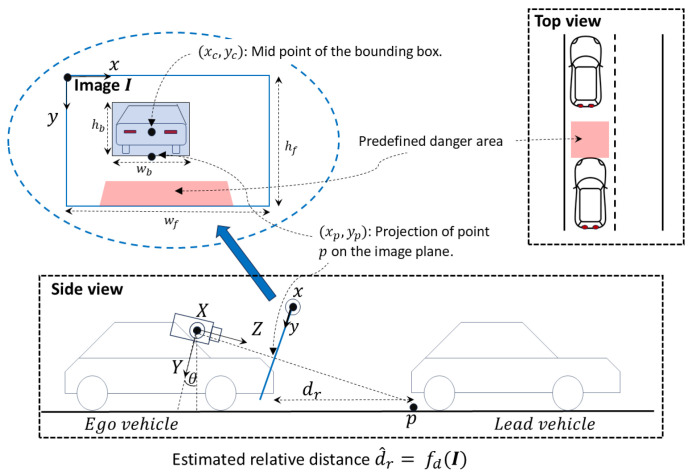
Typical setup for monocular camera-based collision prediction. $d_{r}$ is the relative distance between the ego and lead vehicles. I represents the matrix of pixel values in the image plane (xy). $w_{f},h_{f}$ and $w_{b},h_{b}$ are the dimensions of the full image frame and a detected bounding box, respectively. $f_{d}$ is a function that maps the information contained in I to real-world distance. For example, it could be a function based on camera geometry and calibration that maps the projection $\left(x_{p},y_{p}\right)$ of a point *p* on the ground plane to the corresponding distance $d_{r}$ ([25]) or implemented as a deep neural network that produces depth maps ([26]). Note that in the side view, the image plane (blue line) is shown with its *x*-axis pointing out of the paper. In the top left of the figure, the image plane is shown rotated such that the xy plane is on the paper (enclosed in the blue ellipse). Relative velocity can be estimated as the rate of change of distance, $\hat{v}=\frac{d{\hat{d}}_{r}}{dt}$. TTC is usually computed as $\frac{{\hat{d}}_{r}}{\hat{v}}$.

Most studies use a pre-trained deep learning model (e.g., YOLO [20], SSD [21], and MobileNet [22]) to detect objects in the video stream and then proceed to compute a threat metric based on the detected objects. Figure 3 shows the typical setup used in monocular camera-based collision warning systems, and the notation introduced there will be used in the rest of this section. Different threat metrics have been proposed to assess the risk of a collision, which include,

- relative distance between an obstacle and the ego-vehicle (${\hat{d}}_{r}$),
- relative velocity of an obstacle with respect to the ego vehicle ($\hat{v}=\frac{\hat{dd_{r}}}{d\;t}$),
- time-to-collision (TTC)—typically computed as $\frac{{\hat{d}}_{r}}{\hat{v}}$,
- and predefined areas on the image frame (shaded in red in Figure 3).

One study [23] has used both TTC and relative distance together with other threat metrics. Table 2 provides a summary of the different components used in the threat metric-based pipelines, while Table A1 in Appendix A provides more details.

Systems that use relative distance as the threat metric issue a warning if the estimated distance (${\hat{d}}_{r}$) falls below a predefined threshold value, whereas those that use relative velocity issue a warning if the estimated velocity ($\hat{v}$) is above a threshold. When TTC is used, a warning is issued if it is below a threshold.

Having a single threat metric makes a system less robust to different situations. For example, if the ego vehicle is dangerously close to the lead vehicle but is maintaining an almost zero relative velocity, the TTC will be high and if it is the only metric used, a warning will not be generated. However, even a slight slowing down of the lead vehicle can cause a collision [24]. On the other hand, in slow-moving traffic, vehicles usually move close to the lead vehicle in a relatively safe manner but if distance is used as the sole threat metric, it can generate false warnings. Therefore, multiple threat metrics are needed for accurate predictions in such situations. Interested readers are referred to [24] for a review of collision threat metrics.

Note that in the rest of this paper, unless mentioned otherwise, the video input to a collision warning system is considered to arrive from a single monocular camera.

#### 3.1.1. Relative Distance as the Threat Metric

Deep learning-based depth estimation has been used to estimate the distance to detected objects. Saleh et al. [27] utilize the monocular depth estimator proposed in [28], which consists of a convolutional neural network (CNN)-based encoder-decoder architecture trained with transfer learning. The proposed system generates a warning if the estimated distance is within a predefined risk zone (${\hat{d}}_{r}<15$ m). Wang & Lin [29] propose an unsupervised learning method that integrates depth information with the 2D location of detected objects in the image plane. This method requires binocular image acquisition but does *not* require ground-truth disparity maps and is expected to enhance collision warning capabilities by effectively incorporating depth information into the analysis. However, the computation used to trigger a collision warning based on the estimated depth is *not* mentioned in their paper.

Instead of detecting individual objects, Lai et al. [30] determine the *drivable area* in front of the ego vehicle and its path of interest from the video input. The drivable area is segmented by the multi-task semantic attention network (MTSAN) proposed in the same paper. The path of interest is the main lane occupied by the ego vehicle if lane lines are detected. If not, a straight line path is assumed. Subsequently, they determine the region within the path of interest that is non-drivable. I.e., the intersection of the path of interest and the non-drivable area. Then the closest point in this area (the one closest to the bottom of the image plane) is identified, and the horizontal line across this point is considered the stop line. If the estimated distance to this stop line is less than 15 m, a forward collision warning is issued. Since a monocular camera does not provide explicit depth information, as an approximate solution, they assume the road is flat and use the geometric relation between the road and the camera to estimate the distance. The proposed system also consists of a lane departure warning system.

Some authors have used heuristics based on bounding box dimensions and position to estimate relative distance. Elkholy et al. [31] estimate distance to a detected object based on the width of its bounding box, where the system generates a signal to decelerate the vehicle if the width of a bounding box is between 40% and 60% of the image width, i.e., $0.4w_{f}\leq w_{b}\leq 0.6w_{f}$. They refer to this as a “watch out” scenario. If a bounding box spans more than 60% of the image width, i.e., $w_{b}>0.6w_{f}$, the obstacle is considered extremely close to the ego vehicle (warning scenario) and autonomous braking is applied to its maximum extent. A critical problem with this sort of approach is that the warnings depend not only on the relative distance to a detected vehicle but also on its actual width, which may cause the system to not issue warnings to narrow vehicles (e.g., bikes) despite being close and/or issue false warnings to wider vehicles that are not too close. Joshi et al. [32] partly overcome this in their bounding box width-based distance estimation scheme by specifying different thresholds for different classes of vehicles. These thresholds are set to represent a relative distance of approximately 14 m. For a detected object, they compute a safety factor defined as ${\left(1-w_{b}\right)}^{4}$ [33]. A warning is generated if a vehicle is detected in the same lane as the ego vehicle and the safety factor is below the specific threshold for the detected vehicle class. The mid-point $\left(x_{c},y_{c}\right)$ of the bounding box is used to determine same lane occupancy. However, very limited experiments have been performed to evaluate these two collision warning systems (or their components) with only a few instances (2–6 images) reported and no quantitative evaluation against annotated videos.

Madhumitha et al. [34] on the other hand, estimate relative distance based on the position of the vehicle bounding boxes. More specifically, they extract the center coordinates $\left(x_{c},y_{c}\right)$ of each bounding box and issue a warning if they are large compared to the dimensions of the full image frame, i.e., if $\frac{x_{c}}{w_{f}}\geq 0.2\;AND\;\frac{y_{c}}{h_{f}}\geq 0.7$. While it is intuitive that higher the value of $y_{c}$ the closer an object is to the ego vehicle (assuming that the *y*-coordinate increases from top to bottom of the image as in Figure 3), it is not clear why $x_{c}$ does not have an upper bound in addition to the lower bound to decide on the lateral proximity of a detected object to the right of the ego vehicle. Moreover, an inherent problem in this approach is that the position of the mid-point $\left(x_{c},y_{c}\right)$ depends not only on the distance to an object but also on its size. E.g., a tall vehicle can have a low $y_{c}$ despite being close to the ego vehicle resulting in a missed warning. We also note that although they recognize the importance of the relative speed between the ego vehicle and others, it is not used in the generation of collision warnings according to the aforementioned definition. Furthermore, although they use DeepSORT [35] to track detected objects, it is primarily used to improve the detected bounding boxes and *not* to make predictions about their future location.

Unlike the other methods mentioned in this section, Kabir and Roy [36] use *future* distance as the threat metric. They estimate the relative distance ${\hat{d}}_{r}\left(t_{0}\right)$ and velocity ${\hat{v}}_{r}\left(t_{0}\right)$ between the ego vehicle and an obstacle at the current time $t_{0}$. Using ${\hat{d}}_{r}\left(t_{0}\right)$ and ${\hat{v}}_{r}\left(t_{0}\right)$, the distance ${\hat{d}}_{r}\left(t_{0}+\Delta t\right)$ at a *future* time $t_{0}+\Delta t$ is forecasted through a model based on Newtons’ laws of motion. Δt is determined based on a minimum stopping time and an alarm is activated if two conditions are met, (1) ${\hat{d}}_{r}\left(t_{0}+\Delta t\right)$ falls below a safe stopping distance (3 m in their experiments) and (2) the obstacles’ lateral position, based on the midpoint of its bounding box $\left(x_{c},y_{c}\right)$, is within a pre-calibrated region in the image frame (this region is supposed to approximately align with the region between the left and right edges of the ego vehicle). In this work, object detection is performed using Mobilenet [22], a deep-learning based object detector and classifier. Distance estimation is carried out using two instantaneous images of the object based on pinhole camera geometry. This method of estimating the relative distance and velocity requires tracking an object over at least two consecutive frames. However, the exact method used for object tracking is not mentioned in the paper.

#### 3.1.2. Relative Velocity as the Threat Metric

In addition to detecting objects, these methods need to track the detected objects across frames in order to estimate the relative velocity.

In the method proposed by Ibrahim et al. [37], distances to detected objects are estimated using the height and width of the bounding boxes based on a regression formula [38]. Objects are tracked using a rather simple approach—if the position of an object detected in the current frame is within 5% of that of an object detected in the previous frame, they are considered the same object. Subsequently, the relative speed is computed as the change in distance between the current and previous frames divided by the time between the two frames (Δd^rΔt). A collision warning is triggered when a vehicle shares the same lane as the ego vehicle, and their relative speed exceeds a specified threshold. The effectiveness of the simple tracking method is questionable in complex driving scenarios with occlusions. They report a quantitative value for the accuracy of the relative speed estimation based on a dash-cam video of a real driving scenario. However, the method used to obtain ground-truth values for the relative speed is *not* mentioned. Furthermore, it appears that the evaluation is limited to a single video, details of which are not provided.

Lee et al. [25] estimate the distances to objects using camera geometry and its intrinsic parameters. The change in distance ($\Delta {\hat{d}}_{r}$) between the current and previous frames is also estimated (how the same vehicle is identified between the two frames is not mentioned). A warning is generated if ${\hat{d}}_{r}<10$ m and $\Delta {\hat{d}}_{r}<0$ (positive relative velocity towards the ego vehicle). Their proposed system has cameras to detect objects in front, to the rear, and to the left and right of the ego vehicle.

#### 3.1.3. Time-to-Collision (TTC)

Methods that use TTC also rely on object detection, tracking, and some form of estimating the change in relative distance. Albarella et al. [39] track detected objects using the Global Nearest Neighbour algorithm [40] and a bank of Kalman filters. They then compute the TTC for each tracked object based on the change of its width ($\Delta w_{b}$) in two consecutive frames using a pinhole camera model (details are available in the paper). Note that this TTC estimation does *not* require camera calibration. If the TTC for an object is below a given threshold (~3 s in this case) and if the object is in the path of the ego vehicle, a forward collision warning is generated. In this method, an object is considered to be in the path of the ego vehicle if its bounding box, as predicted by its Kalman filter, is in a pre-calibrated region of the image plane.

Venkateswaran et al. [41] rely on tracking of detected vehicles (using centroid prediction, Hungarian algorithm, and Kalman filtering), and inverse perspective mapping (IPM) [42] to estimate the relative distance and velocity. They use these to estimate how long it would take for the relative distance to reach a predefined minimum value. This is considered an estimate of TTC and a warning is generated if it is below a threshold.

Pyo et al. [43] employ a model that uses the width of the road (the separation between the roads’ reference lines) and the height of the camera to determine the distance to the lead vehicle in the same lane. Using these measurements, the system calculates TTC at intervals of 0.5 s and generates visual warnings for users when a collision is predicted to occur within the next 3 s. However, a distance estimate alone is *not* sufficient to compute the TTC as it requires the rate of change of relative distance. They do *not* precisely state how the estimated distance is used to compute the TTC.

Rill and Farago [44] utilize optical flow and monocular depth estimation (MonoDepth [26]) to determine the speed of the ego vehicle. YOLOv3 [20] is used to detect the lead vehicle. They define TTC as the time it would take for the vehicle to come to a complete stop if the current speed is maintained. A linear regression model is trained to estimate TTC using the ego vehicles’ speed, disparity values from MonoDepth, and the width of the leading vehicles’ bounding box as predictors. Note that this TTC estimation applies only to stationary obstacles as it is based on the ego vehicles’ speed, not the relative velocity between the ego and the lead vehicle. Furthermore, the ground-truth TTC values in their dataset are based on complete stops. Their results also indicate that using the bounding box width as the sole predictor is sufficient to achieve a very similar TTC estimation accuracy to that of the full model (RMSE with respect to ground-truth: 1.05 s vs. 1.02 s).

#### 3.1.4. Multiple Threat Metrics

Using multiple threat metrics to predict possible collisions makes a system more robust to different situations on the road. Zhang et al. [23] base their warnings on relative distance and velocity. They also consider additional factors such as the type of obstacle (e.g., pedestrian or vehicle), its lane (e.g., same or adjacent lane), and the type of environment (structured or unstructured) when issuing warnings. They use a CNN for object detection, the camera geometry to estimate distance to detected obstacles, a multi-object tracking algorithm based on correlation filters [45] to track objects, and the distance estimates of the last five frames to compute the relative velocity.

#### 3.1.5. Threat Evaluation Based on a Predefined Region on the Image Frame

Several methods use a predefined area on the image frame such that if a detected vehicle enters this region, a warning is generated. This area is calibrated to correspond to a predefined longitudinal distance range in front of the ego vehicle within some lateral bounds (depicted by the area shaded in red in Figure 3). With this technique, only object detection is required during real-time inference. However, the main disadvantage is that they ignore the relative velocity, which may lead to missed or false warnings.

**Table 2:** Summary of threat metric-based collision warning pipelines. Refer to Table A1 in Appendix A for more details. A dash “-” in a column indicates that the particular component is not a part of the pipeline of the respective method, whereas “Method not specified” indicates that it is a part of the pipeline but the exact method used is not specified.

	Techniques/Methods Used in Different Components of the Pipeline					Quantitative
**Study**	**Object Detection**	**Relative Distance**	**Object Tracking**	**Relative Velocity**	**Threat-Metric**	**Evaluation of Collision Predictions**
Elkholy et al. (2020) [31]	CNN	Bounding-box width	-	-	Relative distance	No
Joshi et al. (2024) [32]	CNN	Bounding-box width	-	-	Relative distance	No
Kabir & Roy (2022) [36]	CNN	Camera geometry	-	-	Future relative distance	No
Lai et al. (2021) [30]	CNN	Camera geometry	-	-	Distance to closest non-drivable point in the path (CNN-based)	No
Madhumitha et al. (2020) [34]	CNN	Normalized bounding-box center	CNN+Kalman filters	Normalized bounding-box width:height ratio	Relative distance	No
Wang & Lin (2020) [29]	CNN	CNN	-	-	Relative distance	No
Saleh et al. (2021) [27]	CNN	CNN	-	-	Relative distance	No
Lee et al. (2022) [25]	CNN	Camera geometry	-	Method not specified	Relative distance and velocity	No
Ibrahim et al. (2020) [37]	CNN	Camera geometry	Bounding-box proximity	$\frac{\Delta Distance}{\Delta Time}$	Relative velocity	No
Albarella et al. (2021) [39]	CNN	Inverse perspective mapping	Kalman filtering + Hungarian algorithm	Bounding-box scale change	TTC	Yes
Venkateswaran et al. (2021) [41]	CNN	Inverse perspective mapping	Kalman filtering + Hungarian algorithm	$\frac{\Delta Distance}{\Delta Time}$	TTC	No
Pyo et al. (2016) [43]	CNN	Camera geometry	-	Method not specified	TTC	Yes
Rill et al. (2021) [44]	CNN	CNN	-	Optical flow	TTC	Yes
Zhang et al. (2020) [23]	CNN	Camera geometry	Correlational filters	Distance estimates of last 5 frames	TTC + Relative distance and others	No
Lin et al. (2020) [46]	Morphological operations	Camera geometry	Lucas–Kanade optical flow	-	Pre-calibrated region on image plane	No
Wee et al. (2022) [47]	CNN	—	-	-	Pre-calibrated region on image plane	No

Lin et al. [46] proposed a method to generate warnings for forward collisions and overtaking vehicles. Forward collisions are issued when the 2D bounding box of a detected vehicle intersects a predefined region of the image. This region corresponds to the area between the 30 m and 50 m range in front of the ego vehicle (the system is calibrated for this). They also make several assumptions such as the width of a lane is 3–4 m, the velocity of the vehicle is over 60 km/h, the radius of curvature of the lane is over 250 m, and the cameras visible range is over 120 m. We note that their vehicle detection method or other components of the forward collision warning system do *not* really use deep neural networks although convolution masks are used. However, they employ a CNN model (CaffeNet) in their overtaking vehicle detection and warning component, which is the reason for including this study in this review. They provide experimental results for vehicle, motorcycle, lane marking, background, overtaking, and lane change detection but not for forward collision warnings. Wee et al. [47] also issue forward collision warnings for objects detected in front of the ego vehicle that are within a predefined region in the same lane (referred to as the safe driving region but its calibration details are not provided).

**Table 3:** Summary of collision probability-based methods. Please refer to Table A2 in Appendix A for more details and Table 4 for a summary of datasets. Abbreviations: S.atten- Spatial attention, T.Atten- Temporal attention.

Study	Learning	Deep Learning Components					Dataset
	**Method**	**CNN**	**GNN**	**RNN**	**S.Atten**	**T.Atten**	
Strickland et al. (2018) [48]	Supervised	✓	-	✓	-	-	Simulation
Zeng et al. (2017) [49]	Supervised	✓	-	✓	✓	-	DAD
Chan et al. (2017) [50]	Supervised	✓	-	✓	✓	-	A3D
Suzuki et al. (2018) [51]	Supervised	✓	-	✓	✓	-	NIDB
Corcoran et al. (2019) [52]	Supervised	✓	-	✓	✓	-	DAD
Fatima et al. (2021) [53]	Supervised	✓	-	✓	✓	-	DAD
Karim et al. (2022) [54]	Supervised	✓	-	✓	✓	✓	DAD, CCD
Yi et al. (2023) [55]	Supervised	✓	-	✓	✓	✓	AVA
Malawade et al. (2022) [2]	Supervised	✓	✓	✓	✓	-	DoTA, Simulation
Bao et al. (2020) [56]	Supervised	✓	✓	✓	-	-	CCD, A3D
Mahmood et al. (2023) [57]	Supervised	✓	✓	✓	-	-	DAD, CCD, DoTA
Jung et al. (2018) [58]	Supervised	✓	-	-	-	-	Cityscape
Kim et al. (2021) [59]	Supervised	✓	-	-	-	-	YouTube-Crash, Simulation
Bao et al. (2021) [60]	Reinforcement	✓	-	-	✓	-	DAD, DADA
Cho et al. (2023) [61]	Reinforcement	✓	-	✓	✓	-	DADA

### 3.2. Direct Estimation of the Probability of a Future Collision Using Deep Learning Models: Collision Probability-Based Methods

As mentioned before, collision probability-based methods employ deep learning models to predict the probability of a future collision based on the current and some number of past video frames. These models are trained using video datasets containing accident and normal scenes.

We start the review of these methods with those that primarily use CNNs in a supervised learning setting without the involvement of recurrent neural networks (RNNs) or attention mechanisms. We then proceed to more advanced architectures involving RNNs, attention mechanisms, and graph neural networks (GNNs). Towards the end of the section, we review methods that use similar deep learning architectures but in a reinforcement learning setting. Note that although we review these methods under different categories, as shown in Figure 2, they are not mutually exclusive as some techniques are shared between categories. E.g., attention is used in methods that use GNNs as well as those that do not use GNNs. We also observe that some of the deep learning-based methods reviewed here are designed to predict accidents regardless of whether the ego-vehicle is involved in it or not. However, we include them in this review because they are trained and tested on datasets that contain ego-involved accidents (although the fraction of such accidents in some datasets is low as discussed later) and the camera is mounted on the ego vehicle.

#### 3.2.1. Methods Based on Convolutional Neural Networks

**Figure 4:**
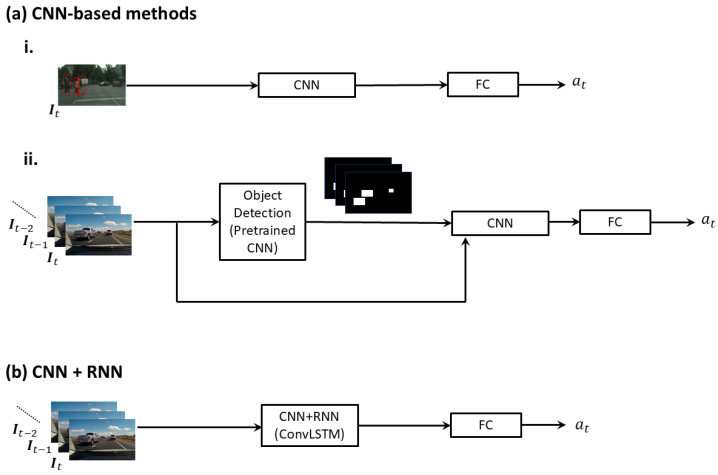
Predicting collisions with (**a**) CNNs (i and ii show the methods proposed in refs. [58] and [59], repetitively.) and (**b**) CNNs combined with RNNs. $I_{t},I_{t-1},I_{t-2},\ldots$ is the sequence of input image frames with $I_{t}$ containing the RGB channels of the image at current time *t*. $a_{t}$ is the predicted probability of a future collision.

Some methods rely on CNNs as their primary model architecture. Jung et al. [58] propose a CNN to predict warning situations involving pedestrians and cyclists, as shown in Figure 4a(i). Unlike traditional approaches that first detect pedestrians and then use a different model to predict potential collisions, the proposed CNN directly predicts warning situations from the input image. Note that they only use the current image frame $I_{t}$ to make the prediction.

Kim et al. [59] also use a CNN to predict future collisions as shown in Figure 4a(ii). They first detect and track objects in the sequence of input image frames. The RGB channels of the input images and the detected bounding boxes (as images) are concatenated to form a 4-channel image sequence that is fed as input to a CNN (simplified version of VGG-16), which predicts whether the detected objects will cause an accident with the ego-vehicle or not. For training the CNN, they use synthetic data generated by a simulator they have developed based on the Grand Theft Auto 5 (GTA V) video game. They note that collision annotations in the synthetic data can be incorrect due to errors in trajectory prediction and refine them using the Constant Turn Rate and Acceleration (CTRA) model or the Constant Curvature and Acceleration (CCA) model [62] for use in the training phase. Furthermore, in the inference phase, real images are transformed into synthetic-like images before being presented to the prediction model. This transformation is learned using a CycleGAN with a feature loss between the synthetic data and a real dash-cam video dataset from YouTube. Final prediction is obtained by averaging the prediction for the real image sequence and that for the transformed sequence. Test results on 122 dash-cam video pairs (one with an accident and the other without an accident in each pair) collected from YouTube by the authors demonstrate small improvements in AUC when the aforementioned annotation refinement and image transformations are used.

#### 3.2.2. Convolutional and Recurrent Neural Networks

Strickland et al. [48] proposed a deep neural network with convolutional long short-term memory (ConvLSTM) layers that takes videos from multiple ego-mounted cameras (dash-cam and a camera on each side mirror), proprioceptive data, and driving commands as input, and predicts the probability that a collision will occur in the future. Note that we include this work in our review as they provide experimental results separately for the three cases where, [camera-only], [camera+proprioceptive state], and [camera + proprioceptive state + driving action] are used as input sources, respectively. Sequences of image frames from the cameras pass through three ConvLSTM [63] layers, which process input images spatiotemporally, and two fully connected layers. The simplified model architecture is shown in Figure 4b. They have performed multiple forward passes through the neural network with dropout (randomly dropping connections in the ConvLSTM units), which provides an estimate for the uncertainty or the confidence of the predicted probability [64]. The proposed model is trained and tested using simulated data generated by the tool V-REP [65], where the accidents had been automatically labeled using the recorded positions of the ego and the other vehicles.

#### 3.2.3. Adding Attention: Spatial and Spatiotemporal

Attention mechanisms attempt to mimic the natural ability of humans to focus on salient regions in complex scenes and disregard irrelevant ones, which allows efficient and effective processing of visual input. In computer vision, attention mechanisms learn to adaptively weight the input features according to their importance based on the input. They have provided advancements in many areas such as image classification, object detection, face recognition, semantic segmentation, and accident prediction, among others. Furthermore, they improve the interpretability of vision systems as well. Attention can be categorized into four basic types based on what dimension the attention masks (weights) are applied to: *Channel attention* to select important channels of information, *spatial attention* to select important regions of an image, *temporal attention* to select important frames in a video along the time dimension, and *branch attention* to select important branches of the network if it has a multi-branch structure. Some methods have combined these types, e.g., *spatial & temporal (spatiotemporal) attention*, and *channel & spatial attention*. We refer interested readers to [66] for a comprehensive survey on attention mechanisms in computer vision.

Collision prediction methods reviewed here have used either spatial or spatiotemporal attention and we review them in the rest of this section.

##### Spatial Attention

**Figure 5:**
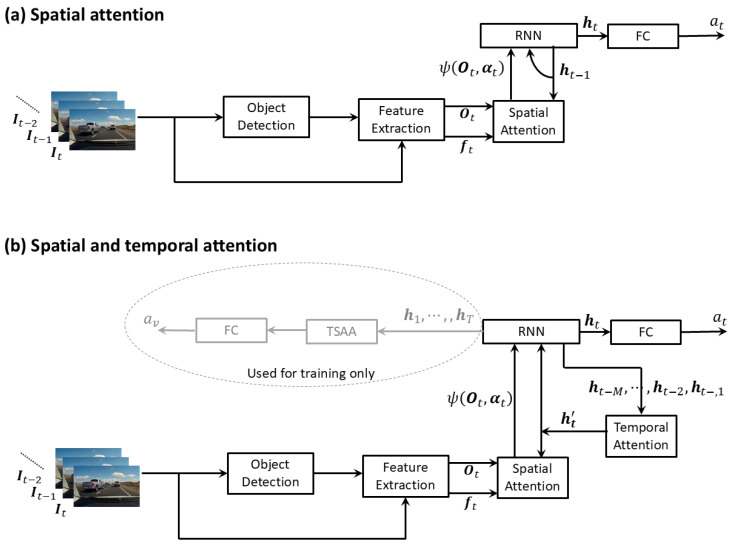
Predicting collisions with (**a**) spatial attention and (**b**) spatial and temporal attention. $O_{t}$ and $f_{t}$ denote the features extracted from the detected objects and the full frame at time *t*, respectively. $\psi (O_{t},\alpha_{t})$ denotes the features aggregated according to attention weights $\alpha_{t}$. $h_{t}$ and $h_{t}^{\prime }$ represent the hidden states of the RNN. $a_{t}$ is the predicted probability of a future collision. More details are presented in the text.

Several methods [50,51,53] make use of spatial attention to focus more on some objects than others. This enables a model to learn to focus on cues such as motion, appearance, and location of risky objects, as well as scene semantics, similar to what human drivers do [50]. Methods using attention have shown improved accuracy compared to baseline models without attention. Furthermore, it makes the model more efficient [60]. As shown in Figure 5a, these methods involve object detection and feature extraction from the detected objects and the full frame at each time step *t*. Features extracted from objects and the full frame are denoted by $O_{t}$ and $f_{t}$, respectively. These features are passed through a spatial attention module, which aggregates the features from different objects using weights ($\alpha_{t}$) that are based on the object features themselves and the previous hidden state ($h_{t-1}$) of a recurrent neural network (RNN). The aggregated features $\psi (O_{t},\alpha_{t})$ are provided as input back to the RNN, which learns to predict the probability of a future accident ($a_{t}$) as computed at time *t*. There are subtle differences in the way different authors have computed the attention weights $\alpha_{t}$ and the aggregated features $\psi (O_{t},\alpha_{t})$.

Chan et al. [50] define the attention weight $\alpha_{t}^{i}$ for object *i* detected at time *t* as

(1) $$\alpha_{t}^{i}=SoftMax_{i}\left\{W^{T}\rho \left(W_{e}h_{t-1}+U_{e}o_{t}^{i}+b_{e}\right)\right\},$$

where $o_{t}^{i}$ is the feature vector of object *i* detected at time *t*, and $W,W_{e},U_{e},b_{e}$ are learnable parameters, and ρ is the hyperbolic tangent function. $SoftMax_{i}$ denotes the softmax operator over *i*. Therefore, the attention weights depend on the object features and the previous hidden state of the RNN. The RNN is implemented with LSTM (Long short-term memory) units. The features of *N* number of objects detected at time *t* are aggregated as a concatenation of the attention weighted sum of object features $o_{t}^{i}$ and the features of the full frame $f_{t}$ as follows.

(2) $$\psi \left(O_{t},\alpha_{t}\right)=\left[\sum_{i=1}^{N}\alpha_{t}^{i}o_{t}^{i};f_{t}\right].$$

Suzuki et al. [51] use the same spatial attention scheme. The differences in their approach compared to that of Chan et al. [50] include the incorporation of deep activation features (DeCAF) [67] in the feature extraction stage and the use of a quasi-recurrent neural network (QRNN) [68] as the RNN for better computational efficiency and stability of the output.

Corcoran et al. [52] too propose a spatial attention mechanism very similar to that in [50]. To compute the attention weight for object *i*, they first transform the object feature vector $o_{t}^{i}$ with a learnable parameter matrix *U* so that $Uo_{t}^{i}$ has the same dimension as the previous hidden state $h_{t-1}$ of the RNN. Note that the number of objects considered is restricted to the 10 top-scoring objects detected, and features from the full-frame are also considered as an additional object. Then, the attention weight $\alpha_{t}^{i}$ is calculated as the dot product between $h_{t-1}$ and $Uo_{t}^{i}$,

(3) $$\alpha_{t}^{i}=SoftMax_{i}\left\{h_{t-1}.Uo_{t}^{i}\right\}.$$

They claim that this simpler dot product provides better results than the more complex transformation used in [50], which involves a linear transform followed by a non-linearity and another linear transform (Equation (1)). However, the main contribution of Corcoran et al. [52] is introducing a separate stream to process temporal information (see Figure 1 in [52]). The input to this second stream is formed with the use of dense optical flow field [69] to capture the information related to the motion of traffic objects across video frames such as relative velocity and trajectory. The optical flow fields are represented as three channels (corresponding to the flow vectors in the *x* and *y* directions of the image and the flow magnitude) and are treated as a three-channel image with values scaled to range from 0 to 255. This three-channel flow field image is then passed through a pipeline, which is the same as that for the first stream that handles the RGB images. The outputs of the two RNNs corresponding to the two separate streams are combined through a fully connected network to predict the risk level of an incident/collision (low, moderate, high, and critical risk) at each frame of the input video.

In the approach taken by Fatima et al. [53], attention weights $\alpha_{t}^{i,j}$ are calculated for every pair of objects (i,j) detected at time *t*, based on their features $o_{t}^{j},o_{t}^{j}$ and the previous hidden state of the RNN $h_{t-1}$ as follows.

(4) $$\alpha_{t}^{i,j}=SoftMax_{j}\left\{A_{t}^{i}{\left(B_{t}^{j}\right)}^{T}\right\},$$

where

$$\begin{array}{cc} A_{t}^{i} & =\rho \left(W_{u}h_{t-1}+W_{\theta }o_{t}^{i}+b_{\theta }\right)\\ B_{t}^{j} & =\rho \left(W_{u}h_{t-1}+W_{\phi }o_{t}^{j}+b_{\phi }\right), \end{array}$$

and $W_{u},W_{\theta },W_{\phi },b_{\theta },b_{\phi }$ are learnable parameters.

The aggregated feature vector $\psi (O_{t},\alpha_{t})$ is obtained as a concatenation of attention-weighted object features and those of the full frame,

(5) $$\begin{array}{cc} \psi (O_{t},\alpha_{t}) & =\left[\frac{1}{N}\sum_{i=1}^{N}z_{t}^{i};f_{t}\right],where\\ z_{t}^{i} & =o_{t}^{i}+\sum_{j=1}^{N}\alpha_{t}^{ij}\left(W_{g}o_{t}^{j}+b_{g}\right), \end{array}$$

and $W_{g},b_{g}$ are learnable parameters.

The work proposed by Zeng et al. [49] can also be considered here although their use of attention is more subtle and not directly related to objects in the scene. Their approach is focused on agents of interest that are identified through online tracking-by-detection. They use faster-RCNN [70] to identify candidates for risky regions on the input image. Features of the agents and the candidate regions are extracted using VGG-16 [71]. In order to aggregate temporal information, the sequence of agent features and positions extracted from the past frames are encoded through an LSTM. A level of risk for each candidate region is computed considering the features of the region, the agent, and the spatial relationship between the agent and the region. The region information is aggregated as a sum of region features weighted by the respective risk level so that more attention is given to riskier regions. The aggregated region information is then concatenated with the aforementioned LSTM-encoded agent features to form what is referred to as a “holistic representation”. The sequence of these holistic representations over the past frames is also encoded by a separate LSTM to capture the temporal information. Then, the accident probability for the current frame is computed based on a concatenation of the LSTM-encoded agent features and the “holistic representation”. Finally, they incorporate a model that can forecast the agents’ position in future frames and use it to forecast the accident probability of future frames. Note that they use trainable weight matrices for transformations at various stages mentioned above.

##### Spatial and Temporal Attention

Some methods use temporal attention in addition to spatial attention as shown in Figure 5b. Not all frames in a video sequence are equally important for predicting accidents as some contain discriminatory information while others may not. Temporal attention allows models to focus on salient frames in the image sequence.

Karim et al. [54] use the same attention weights and aggregated features as Chan et al. [50], shown in Equations (1) and (2), except that a modified version $h_{t-1}^{\prime }$ of the previous hidden state of the RNN is used in Equation (1) instead of $h_{t-1}$ and they use gated recurrent units (GRU) as the RNN. The last *M* hidden states $\left[h_{t-M},\ldots ,h_{t-2},h_{t-1}\right]$ are aggregated as a weighted sum through a *temporal attention* module to obtain $h_{t-1}^{\prime }$. The attention weights are learned from the hidden states $\left[h_{t-M},\ldots ,h_{t-2},h_{t-1}\right]$ as shown below.

(6) $$\begin{array}{c} \beta_{t-1}^{k}=SoftMax_{k}\left\{W_{ta}h_{t-k}\right\},\\ h_{t-1}^{\prime }=\sum_{k=1}^{M}\beta_{t-1}^{k}h_{t-k}, \end{array}$$

where $W_{ta}$ are learnable parameters. This aggregated $h_{t-1}^{\prime }$ is also used as the recurrent feedback to the RNN instead of the usual $h_{t-1}$. They also have another temporal attention module referred to as temporal self attention aggregation (TSAA) that uses the hidden states $\left[h_{1},\ldots ,h_{t},h_{t+1},\ldots ,h_{T}\right]$ corresponding to all *T* frames of input the video. This is used only during the training phase to assist the training of the hidden layers of the RNN. Yi et al. [55] improve the work of Karim et al. [54] mainly in two aspects. First, as computing the loss at the level of individual frames can be noisy, they incorporate a bag-level [72] loss into the loss function. I.e., the videos in each training batch are divided into positive and negative classes. For each video clip, the highest out of the probabilities predicted by the GRU for all frames of the video is considered the probability of an accident for that video clip. Then, the loss function is defined so that minimizing it corresponds to maximizing the difference between accident probabilities for the positive and negative classes. The second modification is that they obtain the predicted probability of an accident from the output of the temporal self attention module present in [54] instead of taking it from the output of the GRU.

#### 3.2.4. Graph Neural Networks

**Figure 6:**
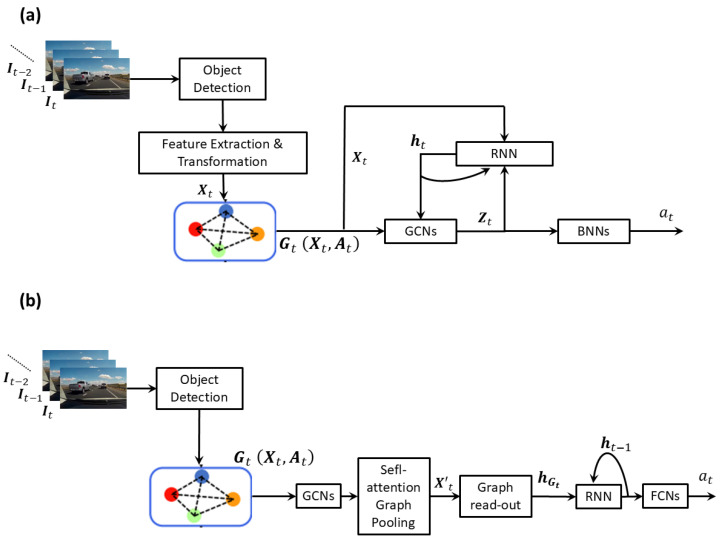
Predicting collisions with graph neural networks (**a**) without attention [56,57] and (**b**) with attention [2]. $G_{t}\left(X_{t},A_{t}\right)$ is a graph defined by node embeddings $X_{t}$ and edge weights $A_{t}$. $h_{t}$ represents the hidden state of the RNN. $a_{t}$ is the predicted probability of a future collision. Definitions of other symbols and more details are provided in the text.

Few authors [2,56,57] have used graph convolutional neural networks (GCNs) [73,74] to capture the spatial relations between detected objects. Main components of such methods are shown in Figure 6.

Bao et al. [56] represent each detected object *i* as a node in a graph whose node feature $x_{t}^{i}$ is computed as a concatenation of object features and full frame features transformed through a fully connected layer,

(7) $$x_{t}^{i}=\left[\phi \left(o_{t}^{i}\right);\phi \left(f_{t}\right)\right],$$

where ϕ represents a fully connected layer. The edge weight $a_{ij}$ between two candidate objects i,j is defined as below so that objects that are closer to each other (in pixel space) receive higher weights.

(8) $$a_{ij}=\frac{exp\left(-d(i,j)\right)}{\sum_{ij}exp\left(-d(i,j)\right)},$$

where d(i,j) is the Euclidean distance between two candidate objects i,j in pixel space. The graph $G_{t}\left(X_{t},A_{t}\right)$, defined with node embeddings $X_{t}=\left[x_{t}^{i}\right]$ and edge weights $A_{t}=\left[a_{ij}\right]$ is passed through a GCN layer. The resulting updated node embeddings are concatenated with the hidden state $h_{t}$ of the RNN and are passed through a second GCN layer,

(9) $$Z_{t}=GCN\left(\left[GCN\left(X_{t},A_{t}\right);h_{t}\right],A_{t}\right).$$

The concatenation with the hidden state $h_{t}$ adds temporal contextual information to the spatial relationships between objects, which has shown improvements in accident anticipation. The hidden state of the RNN, which is a graph convolutional recurrent network (GCRN) [75] in this case, is updated using the current hidden state $h_{t}$ and the output of the second GCN layer $Z_{t}$ concatenated with the combined object and full-frame features $X_{t}$ as follows.

(10) $$h_{t+1}=GCRN\left(\left[Z_{t};X_{t}\right],h_{t}\right).$$

Finally, the output of the second GCN layer $Z_{t}$ is passed through two Bayesian neural network (BNN) [76] layers to predict the probability $a_{t}$ of a future accident. The advantage of using a BNN over a conventional feed-forward NN is that it provides an estimate not only for the probability of an accident but also for the uncertainty associated with the probability. In a BNN, each parameter (weight or bias) $\theta^{i}$ is modeled as being drawn from a Gaussian distribution $N(\mu^{i},\sigma^{i})$ with mean $\mu^{i}$ and standard deviation $\sigma^{i}$ that are learned from the training data. This involves learning the true posterior p(Θ|Data), where Θ includes $\left(\mu^{i},\sigma^{i}\right)$ for all parameters $\theta^{i}$. However, this is intractable in practical problems. Therefore, the variational inference-based approximation referred to as *Bayes by Backprop* [77] has been used to learn an approximate posterior $\hat{p}\left(\Theta |Data\right)$. In the inference phase, for each time *t*, multiple predictions $a_{t}^{1},\ldots a_{t}^{m},\ldots ,a_{t}^{M}$ are made using different networks whose parameters are sampled from $\hat{p}\left(\Theta |Data\right)$. Note that each prediction is a probability vector $a_{t}^{m}={\left(a_{t}^{p,m},a_{t}^{n,m}\right)}^{T}$, where $a_{t}^{p,m}$ and $a_{t}^{n,m}$ are the probabilities of an accident and of no-accident, respectively, which are normalized through a softmax operation. Overall prediction $a_{t}$ is computed as the mean of these multiple predictions,

(11) $$a_{t}=\frac{1}{M}\sum_{m=1}^{M}a_{t}^{m}.$$

The uncertainty $U(a_{t})$ of the prediction is computed as [78],

(12) $$\begin{array}{cc} U(a_{t})= & \frac{1}{M}\sum_{m=1}^{M}\left[diag(a_{t}^{m})-a_{t}^{m}{\left(a_{t}^{m}\right)}^{T}\right]\\ \; & +\frac{1}{M}\sum_{m=1}^{M}\left[(a_{t}^{m}-a_{t}){\left(a_{t}^{m}-a_{t}\right)}^{T}\right]. \end{array}$$

The first part of Equation (12) corresponds to uncertainty due to noise in the data (aleatoric uncertainty), whereas the second part corresponds to model uncertainty (epistemic uncertainty).

Mahmood et al. [57] propose a very similar approach. The main difference is that instead of the visual features of detected objects, they use geometric features of objects. These geometric features are then concatenated with the visual features of the full scene to form the node features of the graph similar to how it is done in [56]. They claim that this combination of geometric and visual features helps mitigate biases in video data and improve generalizability. Experiments performed using three datasets (CCD [56], DAD [50], and DoTA [79]) show higher average precision than [56] when trained on one dataset and tested on another, demonstrating better generalization.

Malawade et al. [2] construct a graph $G_{t}\left(X_{t},A_{t}\right)$, referred to as a scene-graph for each image frame, where the nodes represent the detected objects with features $X_{t}$ and the set of edges $A_{t}$ specifies the pair-wise relations between the detected objects in terms of their proximity, directionality, and the lane occupied (same, left or right lane w.r.t. the ego vehicle). Inverse perspective mapping (Birds-eye view) is used to estimate the proximity between objects. Initially, the node features $X_{t}$ are one-hot encoded vectors and subsequently, they are updated by multiple GCN layers [80]. The final embedding for each node is computed as a concatenation of the embeddings of these multiple GCN layers. Nodes with the concatenated embeddings are passed through a self-attention graph pooling layer, where the irrelevant nodes are filtered out resulting in a graph $G_{t}\left(X_{t}^{\prime },A_{t}^{\prime }\right)$ with a reduced set of nodes $X_{t}^{\prime }$ and edges $A_{t}^{\prime }$. Then, each graph $G_{t}$ is passed through a graph read-out layer that condenses its reduced node embeddings $X_{t}^{\prime }$ to a single spatial embedding $h_{G_{t}}$ (with operations such as mean, max and sum), which is passed through an LSTM to generate a spatio-temporal embedding $z_{t}$ as follows.

(13) $$z_{t},h_{t}=LSTM\left(h_{G_{t}},h_{t-1}\right).$$

Finally, the output of the LSTM, $z_{t}$ is passed through a multi-layer feedforward neural network (FCN) with a LogSoftmax activation after the last layer to produce the confidence score for each class, i.e., collision and no-collision.

#### 3.2.5. Reinforcement Learning-Based Methods

**Figure 7:**
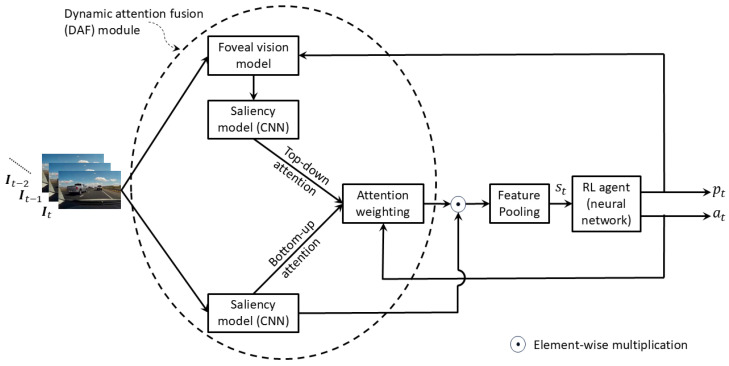
Reinforcement learning approach for collision prediction. $a_{t}$ is the predicted probability of a future collision. $p_{t}$ is the prediction for the drivers’ fixation point.

Bao et al. [60] model the probability of a future collision $a_{t}$ as an action in a Markov decision process (MDP) and train it with reinforcement learning. Figure 7 shows the simplified architecture of their method. The observed state $s_{t}$ at time *t* represents features of the image frame at time *t*. However, since it is inefficient to observe the entire scene, they incorporate a mechanism referred to as dynamic attention fusion (DAF), which consists of a combination of bottom-up and top-down attention in order to focus on the risky region of the image. Bottom-up attention is computed through a CNN-based saliency model [81] that focuses on the salient objects in the scene. Top-down attention is computed by predicting the drivers’ fixation point on the image frame and passing it through a foveal vision module [82] followed by the salience model. To compute the observed state $s_{t}$, the feature volume extracted by passing the input image frame $I_{t}$ through the aforementioned CNN-based saliency model is multiplied by the attention weights produced by the DAF module. The resulting attention-weighted feature volumes are then aggregated through a concatenation of global average and max pooling. The state $s_{t}$ is fed into a deep neural network that learns to predict the probability of a future collision $a_{t}$ and the drivers’ fixation point $p_{t}$ as actions of the MDP. An extended version of the soft actor-critic model [83] is used to train the neural network. The reward for accident anticipation is designed such that for each frame, a correct prediction receives a positive reward but it decays exponentially with time from 1 at the first frame of the video to 0 at the time of the accident. A prediction is considered correct if $a_{t}\geq threshold$ for an accident video and $a_{t}<threshold$ for a normal video. This ensures that early anticipation of an accident receives greater reward. Reward for fixation prediction is based on how close the predicted fixations are to ground-truth fixations provided in the DADA dataset [84].

Cho et al. [61] propose a very similar approach. However, they use the double actor regularized critics [85] instead of the soft actor-critic method of reinforcement learning and achieve more accurate and earlier anticipation of accidents.

### 3.3. Data Sets and Experimental Evaluation

Since collision warning is a safety-critical application, it is essential that any proposed system is thoroughly evaluated using sufficiently large real-world datasets in addition to synthetic data. Collision probability-based methods have used video datasets with annotated collisions/near-collisions to train and test the proposed deep learning models. In contrast, as indicated in Table 2, only two of the threat metric-based studies [39,43] have quantitatively evaluated the accuracy of the generated collision warnings against ground-truth collisions/near-collisions in real or simulated data. Furthermore, we note that except for Albarella et al. [39], there is *no* evidence that any of the systems reviewed in this paper had been tested under protocols similar to those of standards such as Euro or NHTSA NCAP [14,15].

#### 3.3.1. Experimental Evaluation of Threat Metric-Based Studies

Table A1 in Appendix A contains a summary of the experimental evaluation of all the threat metric-based studies included in this review.

Albarella et al. [39] have used both synthetic data as well as real-world data to compare the TTC values predicted by their method against actual values. They have used the CARLA [86] simulator to generate the synthetic data and provide graphs that show how the predicted and actual TTC vary over time for several different conditions. The results show that the predicted TTC is close to the actual value when the actual value is less than about 3 s. Although the predicted TTC is not very accurate for higher values, the proposed system has not resulted in false positives, where warnings were triggered if the predicted TTC is less than 2.1 s. They have performed the real-world experiments using an electric test vehicle following a standard Euro NCAP driving cycle. TTC values obtained through a high accuracy RADAR mounted on the vehicle were used as actual values against which the predictions were compared. Results indicate that predictions are comparable to values obtained through the RADAR and that no false positive nor negative warnings were generated at a TTC threshold of 2.45 s. We note that although graphs showing comparisons between predicted and actual TTC values are provided, *no* numerical measures of the difference between the two are reported. The amount of data used for testing is also *not* mentioned. As for the hardware and processing time, the proposed system has been implemented on an NVIDIA Jetson AGX Xavier Developer board (8 CPU cores, 512 GPU cores and 32 GB of RAM) and is capable of processing video input at 30FPS, which is sufficient for real-time performance.

Pyo et al. [43] have used data collected by themselves on Korean highways during daytime to evaluate the precision of the collision warnings generated by their proposed system. Based on 3140 frames of such data, the system has achieved a precision of 96.36%. However, they have not reported the recall.

As apparent from the above, these studies have used self-collected/generated datasets to evaluate their proposed solutions, which makes direct comparison of results across studies infeasible. The rest of the studies present quantitative evaluations *only* for the sub-components of the pipeline such as object detection and/or distance estimation, but not for the final warnings generated. We do not review their experiments here as previous work in the literature has already reviewed such components (e.g., object detection [87,88], tracking [89], and depth estimation [90,91,92,93]). However, Table A1 in Appendix A presents the results for the evaluation of these sub-components too.

#### 3.3.2. Experimental Evaluation of Collision Probability-Based Methods

Collision probability-based methods have been evaluated using real as well as synthetic dash-cam video datasets. They consist of annotated videos containing collisions/anomalies and videos with normal driving scenes without any collisions, providing positive and negative examples, respectively, to train and test deep learning models. As standard in machine learning, a given dataset is split into training and testing sets of videos. Table 4 shows the real-world datasets used in the studies reviewed here.

**Figure 8:**
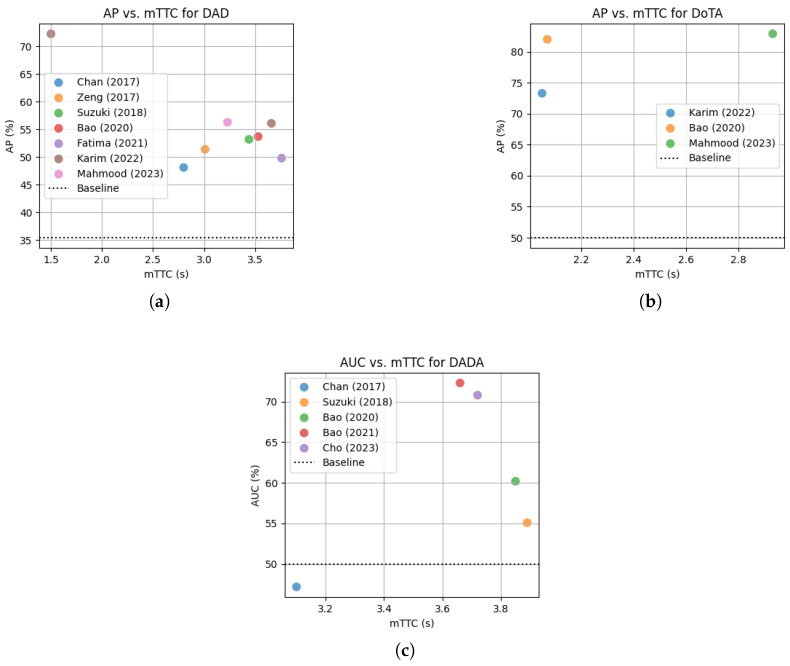
Effectiveness (correctness and timeliness) of collision probability-based methods as measured on popular datasets. More details are available in Table A2 in Appendix A. Note that we did not include the results of the very popular dataset CCD [56] here because as explained in the text, it suffers from video quality bias and all algorithms tested on it have achieved exceptionally good results exploiting this bias. Abbreviations: AP—Average Precision, AUC—Area under the receiver operating characteristics curve, mTTC—mean time-to-collision over different recall values [51]. For a given dataset, baseline AP $=\frac{\#accident\;videos}{\#all\;videos}$ [94], and AUC =0.5. Reference key: Chan (2017) [50], Zeng (2017) [49], Susuki (2018) [51], Bao (2020) [56], Bao (2021) [60], Fatima (2021) [53], Karim (2022) [54], Mahmood (2023) [57], Cho (2023) [61]. (**a**) AP vs. mTTC plot for DAD [50]. (**b**) AP vs. mTTC plot for DoTA [79]. (**c**) *AUC* vs. mTTC plot for DADA [84].

Typical metrics used to evaluate the correctness of predicted collisions include average precision (AP), area under the receiver operating characteristic curve (AUC), precision, recall, and F1-measure. AP is considered a better indicator of a classifiers’ performance on imbalanced datasets [94] than AUC and most studies have used it accordingly. Timeliness of predictions is also crucial to generate warnings to drivers with sufficient time to react. This is typically assessed by mean time-to-collision (mTTC)—also referred to as mean time-to-accident (mTTA). Please refer to [13] for more details on these evaluation metrics. Figure 8 shows the AP, AUC and mTTC values reported on some of the most widely used datasets in the field of collision prediction. Table A2 in Appendix A provides a more detailed account of the experimental results reported by the collision probability-based studies reviewed here.

**Table 4:** Summary of real-world datasets used to evaluate collision probability-based methods.

Dataset	#Accident Videos	#Normal Videos	Length	FPS	Accident Begin Time	Ego-Involved Accidents	Source(s)	Conditions
CCD [56]	1500	3000	5 s	10	Random in the last 2 s.	Yes (53.4%)	YouTube	Weather: rainy, snowy, and good. Lighting: day and night.
DAD [50]	620	1130	5 s	20	At 4.5 s (90th frame)	NA ^a^ (<10%)	Web	Crowded roads
DoTA [79]	4677	Note ^b^	Variable	10	Not fixed	Yes (58.2%)	YouTube	Different countries, weather and lighting conditions.
DADA [84]	2000	None	Variable	30	Not fixed. On average at 5 s.	Yes (~50%)	Crowd-sourced (e.g., YouTube, Bilibili, Youku, Tencent, iQiyi)	Environment: highway, urban, rural, and tunnel. Weather: sunny, rainy and snowy. Lighting: day and night.
A3D [95]	1500	None ^c^	Variable (length 2–20 s)	10	Not fixed	Yes (~60%)	YouTube [96]	Environment: urban, countryside etc. Weather: sunny, rainy and snowy.
YouTube Crash [59]	120	100 ^d^	2 s	NA	End of video	Yes (~100%)	YouTube	Environment/ weather: Various conditions from different countries.
NIDB [97]	4594	1650	100 frames	NA	End of video	NA	Taxis	Lighting: day and night. Environment: Residential, main road, highway. Weather: NA

^a^ The exact number is not available but more than 90% are ego not-involved. ^b^ The pre-anomaly section of each accident video is considered normal. ^c^ Yao et al. [95] have used 230 normal, 10–60 s videos from HEV-I [98] ^d^ 2 s clips from the beginning of the same accident videos.

**The car crash dataset (CCD)** [56] contains 1500 temporally annotated accident videos sourced from YouTube and 3000 normal (non-accident) videos selected from the BDD100K [99] dataset. Each video clip is 5s long at 10FPS. The accident videos span various weather and lighting conditions, and each video is trimmed so that the accident begins within the last 2s. The exact *accident begin time* is randomized across the videos. All algorithms in this review that have been evaluated on CCD achieve an AP of over 99% (baseline is 33.3% as defined in the caption of Figure 8 or Table A2) and an mTTC of over 4.5 s [50,54,56]. However, note that the accident videos in CCD are of much lower video quality than the normal videos selected from BDD100K. The prediction algorithms may learn to discriminate these differences in video quality rather than the properties of accidents and still achieve such high AP values when trained and tested on CCD. To demonstrate this bias, Mahmood et al. [57] show that when the pre-trained GCRNN model [56] (trained on the original CCD accident + high quality BDD100k normal videos) is tested on accident videos from CCD and normal videos from BDD100K but with *quality lowered* to match that of the CCD accident videos, the AP drops drastically from 99.5% to 52.2%. An even worse drop was reported for the DSTA [54] algorithm (AP from 99.6% to 41.3%). Please refer to Table 4 of [57] for more of such experimental results.

**A3D** [95] contains 1500 accident videos of length 2–20 s (at 10FPS) sourced from YouTube, covering different environments and weather conditions. For each video, the anomaly begin and end times were recorded by three human annotators, and these times vary across videos. The begin time is defined as the time at which the annotator feels the accident is inevitable, while the end time is defined as the time when all participants have either come to a complete stop or have recovered normal motion. The dataset contains anomalies involving cars, bikes, trucks, pedestrians, and animals. Two algorithms [50,56] have achieved over 90% AP at an mTTC of over 4 s on A3D. However, it may also be affected by technical differences between accident and normal videos as they are extracted from two different sources (accident videos from YouTube [96] and normal videos from HEV-I [98]). Moreover, even the baseline AP is high at 71.8% for these experiments due to a larger fraction of accident videos compared to normal ones. Another concern is that the subset of A3D used in the said experiments does not include ego-involved accidents [56].

**Near-miss incident database (NIDB)** [97] contains 4594 videos with near-miss incidents and 1650 normal videos, collected in various environments and lighting conditions from cameras mounted on more than 100 Taxis. The near-miss scenes are labeled into six categories: high-bicycle, high-pedestrian, high-vehicle, low-bicycle, low-pedestrian, and low-vehicle. Each video was annotated by three human experts. Scenes with an estimated TTC < 0.5 s were categorized as high risk, while those with TTC > 2 s were categorized as low risk. However, the procedure used by human experts to estimate TTC from videos is not clearly specified. Suzuki et al. [51] have achieved an AP of 99.1% at an mTTC of 4.81 s on NIDB. Nevertheless, NIDB also has a high baseline AP at 73.6% due to its high percentage of accident videos. Furthermore, the percentage of ego-involved accidents is not clearly mentioned although it appears to be high from the descriptions given in [97].

**The dash-cam accident anticipation dataset (DAD)** [50] contains 620 accident and 1130 normal high resolution (720p at 20FPS) videos downloaded from the internet, where the time of accident and the bounding boxes of cars, bicycles, motorbikes and humans are manually annotated. All video clips are 5 s long and the accident begin time is fixed at 4.5 s. The videos are mainly from six cities in Taiwan with crowded roads involving many moving objects, billboards and street signs, complicating the scenes. Furthermore, some objects involved in accidents appear in the video only for a very short period of time. Therefore, DAD is considered a challenging dataset for anticipating collisions [54] with a baseline AP of 35.4%. The highest AP reported on this dataset is 74.3% by Chan et al. [50] themselves. However, the mTTC corresponding to this AP is not reported. Instead, they have reported an mTTC of 1.85 s at recall = 80% and precision = 56.1%. In the experiments performed by Suzuki et al. [51], the AP of the method proposed by Chan et al. was lower at 48.1% but at a higher mTTC of 2.8 s. The next highest AP reported on DAD is by Karim et al. [54], which is 72.3% at an mTTC of 1.5 s. They also report another model with a lower AP of 56.1% but at a higher mTTC of 3.66 s, which provides a better trade-off between correctness and timeliness, as evident from Figure 8a. Note that this dataset has, if any, very few (less than 10%) ego-involved accidents [54]. Therefore, it is not a suitable dataset for evaluating collision warning systems, which try to mitigate ego-involved collisions.

**Detection of Traffic Anomaly (DoTA) dataset** [79] contains 4677 videos (at 10FPS) with annotations for when the anomaly starts and ends (temporal window), where on the video it occurs (spatial localization), and what type of anomaly it is. Each video was reviewed by three human annotators. The anomalys’ start time was defined as the moment it became unavoidable, and the end time was when the involved participants either came to a complete stop or exited the field of view. These times varied across different videos. DoTA dataset has ego-involved (51.9% ego with others + 6.3% ego-only) as well as non-ego anomalies (41.8%), where each type is further divided into 9 categories. Each video is temporally annotated into three segments: normal video prior to the start of the anomaly, window of the anomaly, and post-anomaly. The video segment prior to the anomaly is typically used as negative examples to train accident anticipation models. Mahmood at al. [57] report the best AP of 82.9 at an mTTC of 2.93 on DoTA, while Malawade et al. [2] report an AUC of 78.6 (see Table A2). However, these results are not comparable as they have used different metrics and different subsets of DoTA (a subset of 700/700 positive/negative videos used in [57] and 315/342 positive/negative videos used in [2]).

**The DADA dataset** [84] has been used by Bao et al. [60] and Cho et al. [61] for the evaluation of their reinforcement learning-based algorithms. It consists of 2000 high-resolution videos (1456 × 660p, at 30FPS) representing different road, weather, and lighting conditions, sourced from video websites such as YouTube, Tencent, Bilibili, Youku, and others. It contains both ego-involved and non-involved accidents, which are further divided into a total of 54 sub-categories. For each video clip, the location of the crash-object (i.e., the object involved in the accident) and the *accident window* is identified. The start of the accident window is defined as the time when half of the crash-object appears in the video frame. A possible issue with this definition is that some crash-objects can stay in view for some time without being involved in any anomaly [79]. If the scene returns to normal traffic movement, that time is considered the end of the accident window. In addition to temporal annotations, the DADA dataset also contains fixations maps, fixation duration and saccade scan path to represent driver attention. However, this dataset only has accident videos, and it is not clear what was used as normal (negative) examples to train and evaluate the models in [60,61].

Other real datasets used include YoutubeCrash [59], and AVA [55]. Synthetic datasets used include GTA-crash [59] and the synthetic datasets used in [2]. Table A2 provides a more complete view of the experimental results reported by the reviewed studies.

Four of the above mentioned datasets (CCD, DAD, DADA, and DoTA) have been used by at least two independent studies in their experiments. However, there are challenges that inhibit a thorough comparison between different algorithms. For instance, even on the same dataset, some studies report AP, while others report AUC.

**Figure 9:**
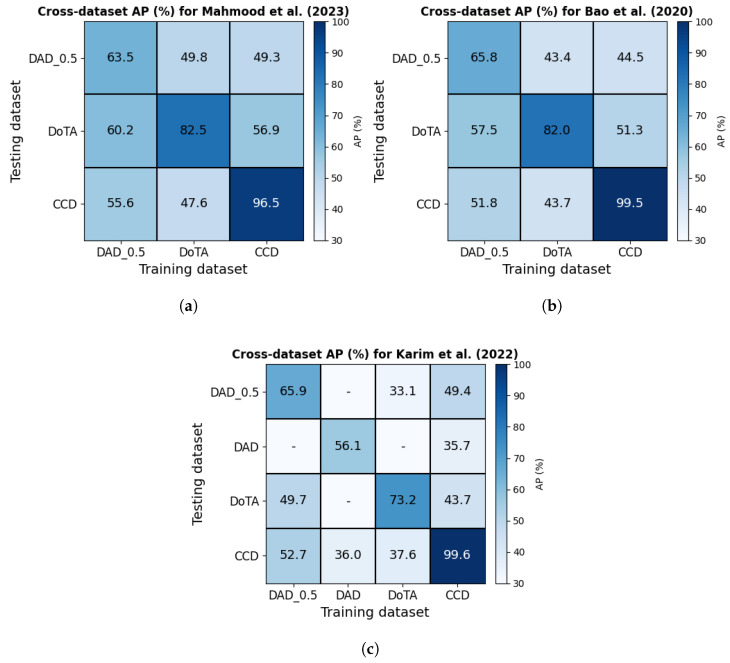
Cross-dataset average precision (AP) for the algorithms proposed by (**a**) Mahmood et al. [57], (**b**) Bao et al. [56], and (**c**) Karim et al. [54]. DAD_0.5 is the balanced version of the DAD with 451 videos in each class used in [57]. Results involving the original DAD are extracted from [54], while all other results are from [57]. Baseline AP for DAD, DAD_0.5, and DoTA are 35.4%, 50%, and 50%, respectively.

Furthermore, there are concerns over the generalizability of these accident prediction algorithms as they have demonstrated poor performance when tested on a dataset different from the one used for training them [54,57]. As shown in Figure 9, cross-dataset evaluations performed in [54,57] reveal that AP on DAD drops down to the baseline level or even worse when trained on other datasets for all algorithms tested. AP on other datasets (DoTA, CCD) also show dramatic drops (by 22–63%) when trained on a different dataset compared to that when trained on a part of itself. The low performance when trained on CCD is already explained by the video quality bias present in it. However, the situation with the other datasets is not so clear. Having said that, training on DAD can result in low AP on other datasets with a high percentage of ego-involved collisions because DAD hardly has any. The drastic drop in AP observed on CCD when the model had been trained on DoTA is hard to assess because the video quality bias present in CCD makes the results of training and testing on CCD itself an inaccurate measure to compare against. Therefore, the true cross-dataset performance is yet to be assessed with different datasets without obvious biases. An additional challenge caused by the use of crowd-sourced dash-cam videos is that they have been captured by different cameras with different intrinsic parameters and they provide different points of view due to variations in the way they are placed in the ego-vehicle. This is referred to as capture bias [57].

Based on the DoTA dataset, which does not have any obvious biases, the GCN-based algorithm proposed by Mahmood et al. [57] achieves the best AP and mTTC compared to [54,56] (Figure 8b). On the other hand, the work by Karim et al. [54] has the best trade-off between AP and mTTC on DAD, closely followed by [56,57] (Figure 8a). Furthermore, ref. [57] achieves better generalization across datasets. According to these results, the work of Mahmood et al. [57] can be considered the most effective algorithm followed by [54,56].

Another major limitation in the experimental evaluation of the proposed collision probability-based methods is that except for [2], the inference speeds of models have *not* been reported, neither on embedded nor other platforms. Therefore, their suitability for real-time collision warning systems remains an open question. Malawade et al. [2] have reported inference speeds of 2071FPS on an Nvidia DRIVE PX 2 embedded platform, and 3923FPS on a PC (AMD Ryzen Threadripper 1950X CPU, 16 GB RAM) with an Nvidia GeForce RTX 2080 Super GPU, respectively. Note that these FPS values are extremely high and we suggest those to be re-assessed.

## 4. Summary and Discussion

Threat metric-based collision warning systems use deep learning techniques in one or more stages of their pipelines to estimate a collision threat metric such as time-to-collision (TTC), relative distance, relative velocity or a predefined region on the image plane. These pipelines consist of object detection, tracking, and/or relative distance/velocity estimation as components. The studies reviewed in this work perform object detection using state-of-the-art deep CNNs as the first component in the pipeline. However, deep learning techniques are not widely used in other components. Object tracking methods used include Kalman filter-based approaches [39,41], Kalman filtering combined with CNNs [34], and the use of simple rules based on proximity [37]. Distance estimation has been performed using either the geometrical relationship between the image plane and the 3D world coordinates [25,30,36,41], deep learning-based depth estimation methods [27,29], or heuristic methods based on the width or position of bounding boxes [31,34]. We note that some of these heuristics are questionable and need to be experimentally validated, which has not been done adequately.

Collision probability-based methods also use CNNs for object detection followed by combinations of other networks, which include RNNS, GNNs and spatial or spatio-temporal attention mechanisms. Instead of computing a threat metric, these networks directly output the probability that a future collision will occur given current and past video frames. While most studies have used a typical supervised learning approach, two [60,61] have used reinforcement learning, although they too rely on labeled datasets for training.

Datasets used to evaluate collision prediction systems include ego-centric driving videos captured by driving a test vehicle, obtained from taxi fleets, dash-cam video clips downloaded from the Internet, and videos generated through simulations. These datasets contain annotated accident/anomaly events and normal driving scenes serving as ground-truth labels for evaluating collision prediction systems and, in the case of collision probability-based methods, for model training as well. Some datasets such as CCD, DAD, DoTA, DADA, YouTubeCrash, and GTACrash are publicly available, allowing different algorithms to be compared on the same data.

However, there are concerns with some of the popular datasets. For instance, video quality bias between accident and normal videos in CCD and a low percentage of ego-involved collisions in DAD. The former is a problem because it is exploited by models to achieve high average precision by simply learning to distinguish differences in video quality instead of those between anomalies and normal scenes, which produces models that do not generalize to other data. The latter is a concern as the primary purpose of collision warning/mitigation systems is to predict ego-involved accidents.

Of the datasets used in the reviewed studies, we suggest DoTA to evaluate collision warning systems as it contains more than 50% ego-involved accidents and pairs of normal and accident videos are extracted from the same set of videos ensuring equal video quality. YouTubeCrash is another dataset with nearly all accident videos containing ego-involved accidents and no apparent technical differences between accident and normal videos, but it has only a limited number of videos (see Table 4).

We observe that the experimental evaluation of the reviewed studies is still at a preliminary stage with some major concerns. First, most of the threat metric-based studies reviewed here have *not* been quantitatively evaluated for their effectiveness using real or synthetic accident data. An exception to this is the work by Albarella et al. [39] who have performed evaluations with both synthetic and real data, including a standard Euro NCAP driving cycle with a test vehicle to evaluate the accuracy of predicted TTC values. The quantity of data used, however, is not reported.

Collision probability-based methods have been trained and tested on labeled accident datasets. However, the results need to be interpreted with caution due to the above-mentioned video quality and accident category biases. Furthermore, experiments have shown very poor generalization of these deep learning models to new data, where the average precision is much lower when the test and train data are *not* splits from the same dataset compared to when they are [54,57]. This is rather surprising since most datasets used are crowd-sourced from the internet and are therefore expected to have a sufficient variety of traffic scenes within them. Hence, we hypothesize that this poor generalization is due to the aforementioned and possibly other *technical* biases in the curation of datasets rather than the novelty of traffic scenes in a different dataset. This also means that although the current cross-dataset experiments have played an important role in revealing such biases, further experiments with unbiased datasets are needed to assess how well these methods generalize to different scenes.

According to AP and mTTC metrics on DAD and DoTA datasets and cross-dataset evaluations, the algorithm proposed by Mahmood et al. [57] demonstrates better performance compared to others. However, note that the inconsistent use of evaluation metrics and utilization of self-collected, non-public datasets hinder comprehensive comparisons between different methods, particularly between threat metric-based and collision probability-based approaches.

In terms of inference speed, many of the threat metric-based methods are capable of running at over 10FPS on embedded hardware platforms and at higher frame rates on systems with more computational power as shown in Table A1 in Appendix A. Such speeds can be considered satisfactory for real-time performance. On the other hand, none of the collision probability-based methods, except the graph neural network-based method proposed by Malawade et al. [2], has been tested for inference speed. This leaves their suitability for real-time applications an open question.

## 5. Challenges and Future Directions

### 5.1. Data

Not surprisingly, the main challenge to the experimental evaluation of collision warning systems is the lack of suitable data. Before we discuss the challenges in acquiring data, it is worthwhile to consider how human drivers learn. We note that human drivers do not learn to anticipate/avoid accidents by getting into many accidents! Instead, they can make use of normal driving experiences to learn the critical area in front of the vehicle, where obstacles should be kept away from [100,101]. They also make use of this experience to predict the trajectories of various objects relative to the ego-vehicle. However, humans are equipped with a visual system that is calibrated through years of routine interaction with the physical world (e.g., walking around obstacles) and fine-tuned to ego-centric road scenes while learning to drive [102,103]. With such experience, trained drivers are able to adapt to a new vehicle by driving it for a short while.

In contrast, most deep learning models need a large number of accident videos (and normal videos too) to learn from. Although many dash-cam accident videos can be obtained through crowd-sourcing, they are from different cameras providing *short* videos of different quality at varying points of view. Therefore, these models cannot benefit from a calibrated camera system. This makes it harder to learn a model that can accurately predict collisions, especially close to the left/right edges of the ego-vehicle as it requires an estimate of the left/right boundaries of the vehicle mapped on to the front-view image plane. This estimation requires a calibrated camera system or it should be learned from a sufficiently long video from the same camera under normal accident-free driving conditions, which are usually not available in crowd-sourced data. Deep learning models have the potential to learn other features from the sequence of video frames that may mitigate this problem, but whether it compensates for the lack of a calibrated camera is yet to be demonstrated with thorough experiments.

Threat metric-based methods on the other hand, are not trained with accident videos and can be calibrated with normal videos captured by a *given* camera system mounted on a *given* vehicle. However, accident videos involving the same vehicle captured from the same camera are needed to evaluate the effectiveness of these methods. This is challenging as one cannot and will not expect a given vehicle to get into many accidents on public roads. A potential solution is to fix dash-cams on a large fleet of vehicles (e.g., taxis), calibrate them, and collect any accidents or near-miss incidents that may arise from these vehicles. Near-miss incident database (NIDB) [51] is such a collection of videos. However, to the best of our knowledge, it is not publicly available and there is no mention of camera calibration.

Another option is to drive a test vehicle with a calibrated camera through standard protocols specified by new car assessment programs (NCAP) such as Euro NCAP [14] and National Highway Traffic Safety Administration (NHTSA) NCAP [15] for testing collision warning and/or automatic emergency braking systems. A caveat is that these protocols involve controlled environments with dummy obstacles and are therefore not as complex as real road scenes.

A possible yet challenging alternative is collecting dash-cam videos of ego-involved accidents from the community, where for each accident clip, sufficiently long normal driving footage from the same vehicle-camera pair is also available. Such normal driving scenes can be used to learn the critical area in front of the ego-vehicle (in the image plane), which the drivers usually keep obstacles away from. Accidents can then be anticipated as predicted violations of this area in the near future.

We also encourage the use of synthetic data in addition to real-world evaluations. Simulators such as CARLA [86], VISTA [104], NVIDIA [105], and V-REP [65] can be used to generate the required synthetic data involving collision and normal scenes. Examples of simulated datasets include Deepaccident [106] and GTA-crash [59].

Since each of the above forms of data collection has its own drawbacks, it is needless to say that testing on multiple datasets acquired through different sources as described above would be the best course of action. However, ego-point-of-view accident videos from some sources such as specific taxi fleets and standardized test drives are scarce to the best of our knowledge. Therefore, we suggest the sharing of such data to facilitate future research. Furthermore, we request authors to report results on multiple evaluation metrics such as AP, AUC, and mTTC along with their respective curves and whenever possible, to use all of the data in a particular dataset so that a fair comparison can be made across different methods.

### 5.2. Incorporating Ttc with Deep Learning

We suggest that collision probability-based methods can be made more useful for advanced driver assistance systems (ADAS) if they are trained to incorporate some indication of TTC in their predictions instead of merely predicting the probability of a future collision without providing any estimate of when the collision might occur. This is important because many ADAS use TTC to assess the severity of a situation and use different thresholds to decide when to trigger warnings, when to activate automatic emergency braking (AEB) and to determine the level of braking force needed if AEB is activated [107]. For example, even for a high probability collision, a warning may be sufficient if 1.5 s < TTC < 3 s. On the contrary, AEB would be required if TTC < 1.5 s. Furthermore, in situations such as turns across paths of oncoming vehicles, accurate TTC estimation assists in evaluating the safety of the maneuver [108].

A promising approach is to model TTC through survival analysis, which can be used to estimate the probability of an event occurring (a collision in this case) within the next *t* seconds [109,110]. This can be modeled as the probability of TTC being less than *t*, i.e., P(TTC<t). Kvamme et al. [109] propose a method to perform time-to-event prediction with neural networks. Some work that has used survival analysis in ADAS research can be found in [111,112,113]. A challenge for modeling TTC through survival analysis as well as the typical probability-based predictions used in existing work is that in most datasets, the time at which the traffic agents involved in the accident start to behave anomalously is not annotated (DoTA [79] is an exception to this). In most datasets, all frames preceding an accident are labeled as positive. However, some of these frames may represent a perfectly normal situation with no abnormalities but the model will be penalized (Note however, that the amount of penalization (the loss) usually decays exponentially with how early a frame is compared to the starting frame of the accident. E.g., see [54].) if these frames are not predicted with a high accident probability during the training phase. Therefore, we suggest that accident datasets are annotated with the time at which the anomalous behavior starts in addition to the time that the accident occurs.

### 5.3. Collision Avoidance in Other Domains

Vision-based collision prediction and avoidance systems have the potential to improve safety beyond typical road traffic environments. Examples include multi-unmanned aerial vehicles (UAVs) and robot navigation [114,115,116]. Distributed control and optimization of multi-UAVs are widely researched areas [117,118], and their safety is critical. In particular, vision-based deep learning methods have been proposed for multi-UAV navigation with collision avoidance [119,120]. We note that many of these systems are currently tested in simulated environments. Therefore, future work should evaluate these systems in real-world environments. A key challenge in using deep computer vision algorithms on UAVs is to develop lightweight models to achieve real-time performance on highly resource-constrained platforms onboard UAVs. Deep learning has also been applied for solely vision-based obstacle avoidance in robot navigation [121,122]. Yet again, how models trained on simulation environments translate into complex real-world scenarios remains to be further explored in future work.

## 6. Conclusions

In this paper, we systematically reviewed vision-based collision warning systems and answered the following questions.

**What are the deep learning methods used and how are they integrated with the various components of a system?**

Deep learning techniques have been used in vision-based collision warning systems to either directly predict the probability of a future collision from video frames (collision probability-based methods) or predict by first computing a threat metric (threat metric-based methods). In both types, pre-trained CNNs have been used for object detection as the first step. In collision probability-based methods, detected objects and their features are passed through another neural network to predict the probability of a collision. RNNs, GNNs, and spatial and/or temporal attention mechanisms have been used to form the prediction model. In threat metric-based methods, object detection is typically followed by distance estimation and/or object tracking to compute a threat metric. Some systems use CNNs in these components while others use more traditional approaches such as camera geometry and Kalman filtering, respectively.

**What are the datasets used and their properties?**

Ego-centric videos captured from test vehicles, taxis, and dash-cam videos downloaded from the Internet have been used. In most such datasets, each video clip is annotated to indicate whether or not it contains an accident/anomaly (some datasets contain additional information too). These annotations serve as ground-truth for model evaluation and/or training purposes.

**What are the methods used in the experimental evaluation and the results achieved?**

The correctness and the timeliness of predictions made by collision probability-based methods have been quantitatively evaluated against ground-truth labels present in real datasets. However, various biases present in some of the most widely used datasets raise concerns over the reliability and generalizability of the results. Furthermore, these methods have *not* been evaluated for inference speed (except for one study [2]).

Threat metric-based methods show reasonable real-time inference speeds. However, in most such studies reviewed here, the correctness of the predictions is *not* quantitatively evaluated using real or synthetic datasets.

Therefore, we conclude that further experiments with carefully curated datasets are needed to assess the effectiveness of vision-based collision prediction/warning systems mounted on the ego-vehicle. Inference speed of the proposed deep learning models used in collision probability-based methods also needs to be assessed on embedded hardware platforms.

## 7. Limitations

This work reviewed only the collision warning systems published in academic literature. However, there are many proprietary systems fitted in modern commercial vehicles. We are unable to review them as their details are not publicly available.

## Figures and Tables

**Table 1:** Main symbols and abbreviations used. Note that symbols will be sub-scripted with respect to time *t* and super-scripted with respect to object ID *i* as needed.

Symbol/Abbreviation	Description
*I*	Input image frame
$d_{r}$	Relative distance between ego and lead vehicles
$v_{r}$	Relative velocity between ego and lead vehicles
TTC	Time-to-Collision
$a_{t}$	Probability of a collision as predicted at time *t*
*O*	Features extracted from detected objects
*f*	Features extracted from the full input frame *I*
*h*	Hidden state of an RNN
α	Attention weights of objects *O*
β	Temporal attention weights of hidden states *h*
W,U and *b*	Learnable weights and biases, respectively
G(X,A)	A graph defined by node embeddings *X* and edge weights *A*
SoftMax	Softmax operator
BNN	Bayesian Neural Network
CNN	Convolutional Neural Network
FC	Fully-Connected layers
GNN	Graph Neural Network
GCN	Graph Convolutional Neural Network
RNN	Recurrent Neural Network
LSTM	Long Short-Term Memory
ConvLSTM	Convolutional Long Short-Term Memory
RL	Reinforcement Learning

## Data Availability

No new data were created thourgh this research.

## Supplementary Materials

The following supporting information can be downloaded at: https://www.mdpi.com/article/10.3390/jimaging11020064/s1.

## Appendix A

Supplementary information is provided in this Appendix A.

**Table A1:** Summary of threat metric-based collision warning pipelines. Abbreviations: EMB—Embedded (hardware platform), mAP—Mean Average Precision, mIOU—mean intersection over union, RMS—Root mean Square, R.—Relative.

Study and Threat Metric	Components of the Pipeline	Dataset(s) Used	Results of Comparison with Ground Truth	Hardware & Inference Speed
Elkholy et al. [31] R.distance	Obj Detection: MobileNet V1 [22]	Simulated dataset generated with Udacity	NA	NA
	Distance estimation: Object width		NA	
	Collision warning		Two sample images	
Joshi et al. [32] R.distance	Obj Detection: MobileNetV2 [123]	Indian driving dataset	NA	NA
	Distance estimation: Bounding box width [33]		NA	
	Collision warning		Few sample images	
Kabir and Roy [36] Future R.distance	Obj Detection: MobileNet [123]	Model based on COCO [124]. Tested with Real self-collected data	Detection accuracy vs. distance plots	Raspberry pi 4 Model B (EMB): 11FPS
	Tracking: not specified	Not evaluated	NA	
	Future R.distance estimation	Real self-collected data	NA	
	Collision warning	Few self-collected instances with a mannequin	Few sample images	
Lai et al. [30] Distance to closest non-drivable point in the path	Obj Det: based on U-Net [125]	Cityscape [126] validation set BDD [99] validation set	mAP = 39.78% mAP = 28.8%.	NVIDIA Jetson Xavier (EMB): 10FPS, Texas Instrument TDA2x (EMB): 15FPS. Both at 512 × 256 input resolution. NVIDIA Maxwell Titan X: 33FPS at 1024 × 512 input resolution
	Drivable area segmentation: based on SSD [21]	Cityscape [126] test set	mIOU = 70.17%.	
	Distance estimation: Camera geometry	NA	NA	
	Lane departure warning: Self proposed	Highway-driving videos captured in Taiwan.	Recall = 98.31% (58/59), Precision = 96.55% (56/58)	
	Collision warning		Few sample images	
Madhumitha et al. [34] R.distance	Obj Detection: SSD512 [21] Tracking: DeepSORT [35] Collision warning	Dash-cam accident videos from YouTube.	Few sample images NA Few sample images	NA
Wang & Lin [29] R.distance	Obj Detection: RefineDet [127]	PASCAL VOC [128] KITTI [129] BDD100k [99]	mAP = 79.75 mAP = 60.10 mAP = 22.91	NVIDIA GTX 1080: 25FPS at 320 × 320 input resolution
	Depth estimation: Modified version of MonoDepth [26]	BDD100k [99]	RMS = 4.85, $\delta_{1}$ = 0.93, $\delta_{3}$ = 0.99, abs-rel = 0.08, sq-rel = 0.925, Log-rms = 0.16, D1-all = 12.48. Note ^1^	
	Collision warning	NA	NA	
Saleh et al. [27] R.distance	Obj Detection: YOLOv3 [20]	MS COCO [130]	mAP @50IoU=58%	NA
	Depth estimation: CNN-based [28]	KITTI [129]	RMS = 4.170, $\delta_{1}$ = 0.886, $\delta_{1}$ = 0.986, rel = 0.093, $log_{10}$ = 0.171.	
		NYUv2 [131]	RMS = 0.390, $\delta_{1}$ = 0.895, $\delta_{3}$ = 0.996, rel = 0.103, $log_{10}$ = 0.043.	
Lee et al. [25] R.distance+ R.Velocity	Collision warning	Real sefl-collected data	One sample image	PC with GPU: 47FPS, NVIDIA Jetson TX2 (EMB): 3FPS. (These frame rates are for object detection only)
	Obj Detection: MobileNetV2 [123]	Self-collected diverse day-night datasets from various perspectives	Precision: 93.0–99.0%, Recall: 93.0–99.0%, Accuracy: 92.9–99.0%.	
	Distance estimation: Camera geometry		Precision: 80.2–89%, Accuracy: 79.6–89%	
	Collision warning		Few sample images	
Ibrahim et al. [37] R. velocity	Obj Detection: YOLO [20]	A single video recorded using a monocular camera installed on an actual vehicle obtained from the internet.	NA	NVidia Jetson TX2: 23FPS
	Tracking: Bounding-box proximity [37]		NA	
	Depth estimation: Camera geometry		Accuracy up to 80.4%	
	Lane Assignment		Accuracy up to 93%	
	R.Velocity estimation: Δdistnce/Δtime		Accuracy up to 80.4%	
	Collision warning		NA	
Albarella et al. [39] TTC	Obj Detection: YOLO [20]	Not evaluated separately	NA	NVIDIA Jetson AGX Xavier (EMB): 30FPS
	Tracking: Kalman filtering + Hungarian Algorithm [132]			
	R.velocity and TTC estimation: Bounding-box scale change	Synthetic+Real self-collected data	Predicted vs. Actual TTC graphs for 3 different instances	
	Collision warning		Warnings based on actual vs. predicted TTC for the above 3 instances	
Venkateswaran et al. [41] TTC	Obj Detection: YOLO [133]	Caltech 99/2001, TU Graz-02, LISA-Q	Precision: 85–99%, Recall: 79–100%	PC (32 core AMD CPU, 64GB RAM) with an Nvidia 2060RTX series GPU: 15FPS
	Tracking: Kalman filtering + Hungarian Algorithm	Real self-collected data	RMSE of detected vs. predicted centroids: 6.45–9.14	
	R. distance: Inverse Perspective Mapping R.Velocity estimation: Δdistnce/Δtime		Few measurements showing a linear relationship between R.distance and the inverse perspective mapped pixels	
	TTC and Collision warning	Not evaluated	NA	
Pyo et al. [43] TTC	Obj Detection: CNN	CalTech 99 [134]	Recall = 100%	Intel i5 3.4 GHz CPU: 50FPS
	Lane detection: Hough Transform	NA	NA	
	Distance estimation: Camera geometry	NA	NA	
	Collision warning	Real self-collected data (daytime, highway)	Precision = 99.36%	
Rill et al. [44] TTC	Obj Detection: YOLO [20]	Real self-collected data	NA	NA
	R.distance: MonoDepth [26]		NA	
	R.Velocity: Optical flow + estimated depth		NA	
	TTC		RMSE compared ground-truth: 1.03 s	
Zhang et al. [23] R. distance & TTC & other factors	Obj Detection: YOLO [20]	Real self-collected data	Few sample images	NA
	Tracking: Correlation filters		NA	
	R. distance: Camera geometry R.Velocity estimation		Few values provided but *not* compared with ground-truth	
	Collision warning		Few sample images	
Lin et al. [46] pre-calibrated region of the image	Obj Detection: morphological operations	Real self-collected data	Precision = 100%, Recall = 62.4% for front vehicle detection.	A PC (4.0 GHz Intel i7 CPU) with Nvidia GTX 1080 GPU: Speed NA
	Tracking (for overtaking): Lucas–Kanade optical flow		NA	
	Lane Change Detection		Precision = 97.9%, Recall = 96.8%	
	Overtaking Vehicle Identification		Precision = 92.5%, Recall = 98.2%	
	Collision warning		NA	
Wee et al. [47] pre-calibrated region of the image (safe driving region)	Obj Detection: YOLACT [135] and a few other models	COCO pre-trained models	mAP = 29.5 for YOLACT Resnet-101	Nvidia GTX 1050 TI: 5FPS, Nvidia Tesla K80: 5FPS (these values are for object detection and lane detection tested separately).
	Lane detection	Real self-collected data	mAP = 24.56 for YOLACT Resnet-101	
	Collision warning	Self-collected real-time input videos	Few sample images	

^1^ Please refer to [136,137] for definitions of evaluation metrics for depth estimation.

**Table A2:** Summary of experimental results of the collision probability-based methods. A citation next to a value indicates that the particular value is from the cited study, not the original study. Similarly, a citation next to values enclosed by parenthesis indicates that all those values are from the cited study. Baseline AP $=\frac{\#accident\;videos}{\#all\;videos}$ [94]. Abbreviations: ACC—accuracy, AP—Average Precision, AUC—Area under the receiver operating characteristics curve, MCC—Matthews correlation coefficient, mTTC—mean time-to-collision over different recall values [51], S.—spatial, ST.—spatial and temporal. RAE—Regularized AutoEncoder.

Study	Techniques Used	CCD (Baseline AP = 33.3%)	DAD (Baseline AP = 35.4%)	DADA (Baseline AP NA)	DoTA ^***a***^ (Baseline AP ≈ 50%)	Other
Strickland et al. (2018) [48]	ConvLSTM	-	-	-	(ACC = 48.9, AUC = 47.2, MCC = −0.04) [2]	Simulated data from V-REP: 3 cameras: ACC = 81.95, MCC = 0.64 dashcam-only: ACC = 70.9, MCC = 0.43
Zeng et al. (2017) [49]	CNN+RNN+ S.Attention	-	AP = 51.4, mTTC = 3.01 s	-	-	-
Chan et al. (2017) [50]	CNN+RNN+ S.Attention	(AP = 99.6, mTTC = 4.52 s) [54]	AP = **74.3**, mTTC = NA Precision = 56.1, Recall = 80.0 $TTC_{80R}=1.85$ s (AP = 48.1, mTTC = 2.8 s) [51] (AP = 48.1, 1.34) [53] (AUC = 71.6, mTTC = 1.17 s) [60]	(AUC = 47.2, mTTC = 3.1 s) [60]	-	A3D [95]: (AP = 93.4, mTTC = 4.41 s) [56]
Suzuki et al. (2018) [51]	CNN+QRNN+ S.Attention	-	(AP = 53.2, mTTC = 3.44 s) [54] (AP = 52.3, mTTC = 3.43 s) [54] (AUC = 58.1, mTTC = 2.22 s) [60]	(AUC = 55.1, **mTTC = 3.89 s**) [60]	-	NIDB [97]: AP = 99.1, mTTC = 4.81 s (baseline AP = 73.6)
Corcoran et al. (2019) [52]	CNN+RNN+ S.Attention	-	ACC = 84.9 ^*b*^	-	-	-
Fatima et al. (2021) [53]	CNN+RNN+ S.Attention	-	AP = 49.8 ^*c*^, **mTTC = 3.76 s**	-	-	-
Karim et al. (2022) [54]	CNN+RNN+ ST.Attention	AP = 99.6, mTTC = 4.87 s	AP = 56.1, mTTC = 3.66 s AP = 72.3, mTTC = 1.5 s $TTC_{80R}=1.81$ s Note ^*d*^	-	(AP = 73.3, mTTC = 2.05 s) [57]	AVA freeway: ACC = 54.5, F1 = 50.4 AVA Road: (ACC = 62.3, F1 = 43.8) [55]
Yi et al. (2023) [55]	CNN+RNN+ ST.Attention	-	-	-	-	AVA freeway: ACC = 63.0, F1 = 61.4 AVA Road: ACC = 70.1, F1 = 61.2
Malawade et al. (2022) [2]	CNN+GCN+ S.attention+RNN	-	-	-	ACC = 70.1, AUC = 78.6, MCC = 0.40. Note ^e^	271-Syn: ACC = 88.1, AUC = 94.6 MCC = 0.54 306-Syn: ACC = 83.7, AUC = 90.9 MCC = 0.68 1043-Syn: ACC = 91.0, AUC = 94.8 MCC = 0.54
Bao et al. (2020) [56]	CNN+GCN+ RNN+BNN	AP = 99.5, mTTC = 4.74 s	AP = 53.7, mTTC = 3.53 s $TTC_{80R}=1.56$ s (AUC = 66.0, TTA = 0.92 s) [60]	(AUC = 60.2, mTTC = 3.85 s) [60]	(AP = 82.0, mTTC = 2.07 s) [57]	A3D [95] ^*f*^ and HEV-I [98]: AP = 94.4, mTTC = 4.92 s (baseline AP = 71.8%)
Mahmood et al. (2023) [57]	CNN+GCN+RNN	AP = 99.6, mTTC = 4.79 s	**AP = 56.3, mTTC = 3.23 s**	-	**AP = 82.9, mTTC = 2.93 s**. Note ^*g*^	-
**Study**	**Techniques used**	**CCD**	**DAD**	**DADA**	**DoTA**	**Other**
Jung et al. (2018) [58]	CNN	-	-	-	-	A part of Cityscape [126] ^*h*^: TPR = 71, FPR = 15
Kim et al. (2021) [59]	CNN	-	-	-	-	YouTubeCrash: AUC = 87.3 GTACrash: AUC = 90.9 GTACrash+YouTubeCrash ^*i*^: AUC = 92.0, $TTC_{80R}\approx 1.3$ s
Bao et al. (2021) [60]	RL with CNN + S.Attention + RAE	-	AUC = 93.8, TTC = 2.78 s ^*j*^	**AUC = 72.3**, mTTC = 3.66 s	-	-
Cho et al. (2023) [61]	RL with CNN + S.Attention + LSTM	-	-	AP = 86.2, AUC = 70.8, mTTC = 3.72 s	-	-

^a^ Results presented in [2,57] are not comparable against each other as they use different subsets of videos from DoTA; ^b^ This is accuracy over four classes of risk: low, moderate, high, critical; ^c^ Fatima et al. refer to the DAD dataset as SA dataset; ^d^ Karim et al. provide results for both high and low AP models for the DAD dataset; ^e^ Results are based on 315 accident and 342 normal videos; ^f^ Have used only 587 of the 1500 accident videos containing only non-ego-involved accidents.; ^g^ Results are based on 700 accident and 700 normal videos; ^h^ Manually annotated by Jung et al. for possible collisions involving pedestrians; ^i^ Trained with GTACrash and tested with YouTubeCrash with domain adaptation; ^j^ TTC is based on a probability threshold of 0.5.
