# Supplementary material for: Vision-Based Collision Warning Systems with Deep Learning: A Systematic Review
Source: J Imaging. 2025 Feb 17;11(2):64. doi: 10.3390/jimaging11020064 (PMC11856197; doi:10.3390/jimaging11020064)
Supplement: Supplementary file 1 [file jimaging-11-00064-s001.zip › Risk of Bias Analysis-PROBAST.pdf]

Online Resource 1 - Vision-based Collision Warning Systems with Deep Learning: A Systematic Review

Prediction Model Risk of Bias Assessment (PROBAST)

	1	2	3	4	5	6	7	8	9	10	11	12	13	14	15	16	17	18	19	20	21	22	23	24	25	26	27	28	29
**Study:**	Elkholy et al. (2021)	Kabir and Roy (2022)	Lai et al. (2021)	Madhumitha et al. (2020)	Wang & Lin (2020)	Saleh et al. (2021)	Lee et al. (2022)	Ibrahim et al. (2020)	Albarella et al. (2021)	Venkateswaran et al. (2021)	Pyo et al. (2016)	Zhang et al. (2020)	Lin et al. (2020)	Wee et al. (2022)	Strickland et al. (2018)	Zeng et al. (2017)	Chan et al. (2017)	Suzuki et al. (2018)	Corcoran et al. (2019)	Fatima et al. (2021)	Karim et al. (2022)	Yi et al. (2023)	Malawade et al. (2022)	Bao et al. (2020)	Mahmood et al. (2023)	Jung et al. (2018)	Kim et al. (2021)	Bao et al. (2021)	Cho et al. (2023)
**Signaling question**																													
**Domain 1: Data instances (video segments)**																													
Were appropriate data sources used (eg, cohort, RCT	PN	PY	PY	PY	PN	PY	PY	PN	PY	PN	PY	PY	PN	PY	PY	PY	PY	PY	PY	PY	PY	PY	PY	PY	PY	PY	PY	PY	PY
Were all inclusions and exclusions of instances appro	PY	PY	PY	PY	PY	PY	PY	PY	PY	PY	PY	PY	PY	PY	Y	Y	Y	Y	Y	Y	Y	Y	Y	Y	Y	Y	Y	Y	Y
**Domain 3: outcome**																													
Was the outcome determined appropriately?	N	N	N	N	N	N	N	N	PY	N	PY	N	N	N	Y	Y	Y	Y	Y	Y	Y	Y	Y	Y	Y	Y	Y	Y	Y
Was a prespecified or standard outcome definition use	N	N	N	N	N	N	N	N	Y	N	N	N	N	N	Y	Y	Y	Y	Y	Y	Y	Y	Y	Y	Y	Y	Y	Y	Y
Was the outcome defined and determined in a similar	N	N	N	N	N	N	N	N	Y	N	N	N	N	N	Y	Y	Y	Y	Y	Y	Y	Y	Y	Y	Y	Y	Y	Y	Y
**Domain 4: analysis**																													
Was there a reasonable number of instances with the	N	N	N	N	N	N	N	N	PY	N	PN	N	N	N	PY	PY	PY	PY	PY	PY	PY	PY	PY	PY	PY	PY	PY	PY	PY
Were all instances included in the analysis?	PN	PN	PN	PN	PN	PN	PN	PN	PY	PN	PN	PN	PN	PN	Y	Y	Y	Y	Y	Y	Y	Y	Y	Y	Y	Y	Y	Y	Y
Were relevant model performance measuresevaluated	N	N	N	N	N	N	N	N	PY	N	N	N	N	N	PN	Y	PY	Y	PN	Y	Y	PN	PN	Y	Y	PN	PY	Y	Y
Were model overfitting and optimism in modelperform	N	N	N	N	N	N	N	N	N	N	N	N	N	N	N	N	N	N	N	N	PY	N	N	N	PY	N	N	N	N

Note: Signalling questions regarding predictors (mainly in domain 2) were ignored since the input to the studies reviewed here are image sequences without any predefined predictors.
